# Structural and functional brain abnormal alteration in patients with type 2 diabetes mellitus: A coordinate-based meta-analysis

**DOI:** 10.1038/s41398-025-03488-z

**Published:** 2025-08-06

**Authors:** Di He, Zeqi Hao, Mengqi Zhao, Mengting Li, Na Hou, Yang Yu, Lulu Cheng, Xize Jia

**Affiliations:** 1 School of Information and Electronics Technology, Jiamusi University, Jiamusi, China; 2 School of Psychology, Zhejiang Normal University, Jinhua, China; 3 School of Foreign Studies, China University of Petroleum (East China), Qingdao, China; 4 Psychiatry Department, the Second Affiliated Hospital Zhejiang University School of Medicine, Hangzhou, Zhejiang China

**Keywords:** Diagnostic markers, Biomarkers

## Abstract

**Background:**

Type 2 diabetes mellitus (T2DM) is a prevalent chronic condition associated with a range of abnormalities in both the functional and structural aspects of the brain. However, existing studies have produced inconsistent results due to disease heterogeneity and small sample size. Therefore, we aim to examine common functional and structural alterations in patients with T2DM.

**Method:**

We searched PubMed, Web of Science, and Embase (published before July 2023) and included a total of 52 valid studies (58 datasets), which have 2160 patients with T2DM and 2124 healthy controls (HCs). Then, we used the anisotropic effect size seed-based *d* mapping (AES-SDM) to examine changes in neural activity and grey matter volume (GMV) in patients with T2DM. To validate the findings of the AES-SDM results, two additional meta-analyses were performed using activation likelihood estimation (ALE) and multilevel kernel density analysis (MKDA).

**Results:**

The results showed that patients with T2DM exhibited abnormal neural activity, functional connectivity of default mode network, and GMV in brain regions related to visual processing, such as the occipital lobe, lingual gyrus, and Heschl’s gyrus. In addition, functional or structural alterations were also found in other sensory-motor, cognitive, and attention-related brain regions in patients with T2DM. The results of ALE and MKDA fundamentally confirmed the findings of the AES-SDM analysis.

**Conclusion:**

These findings offer robust neural markers and deepen our understanding of the neurobiological underpinnings of T2DM.

## Introduction

Type 2 diabetes mellitus (T2DM) is one of the most prevalent metabolic diseases in the world and its incidence is still increasing globally [1, 2]. T2DM is primarily attributed to insufficient insulin secretion and insulin resistance, leading to elevated blood glucose levels [3], which affects the ability to conduct nerve signals and process information, and subsequently affects the patients’ many areas of functioning, including cognitive, mood, awareness, and behavior [4, 5]. In recent years, more and more studies have shown that alterations in brain function and structure are related to the pathological mechanisms of T2DM, suggesting that it is important for us to explore the neural mechanisms of T2DM in depth with the help of neuroimaging tools.

Resting-state functional magnetic resonance imaging (rs-fMRI), which measures spontaneous brain activity by detecting low-frequency blood oxygen level-dependent (BOLD) signal changes, is an important tool for exploring the mechanisms of T2DM [6, 7]. Functional connectivity (FC) is one of the most commonly used methods for rs-fMRI, which is a measure of synchronized activity between different brain regions that helps us to understand the exchange of information and synergy between brain regions [8, 9]. In most of the available studies, FC changes in patients with T2DM have been explored to find evidence of altered neural networks in patients with T2DM [10, 11]. Among the many brain networks, the default mode network (DMN), one of the most important resting-state brain networks, is characterized by high activity when the brain is not engaged in specific tasks and low activity when the brain is focused on the external environment [12]. Marder et al. [13] found that people with T2DM exhibited reduced FC in DMN, and were associated with an increased risk of cognitive impairment. However, FC can only respond to the correlation of activity between brain regions and does not directly identify which brain region is at fault [14]. In contrast, local metrics can examine brain areas with abnormal spontaneous neural activity, and the combination of FC and local metrics can help to assess the functional state of the brain more comprehensively and provide more clues for the diagnosis and treatment of T2DM [15–18].

In the analysis of regional spontaneous brain activity, three common local metrics are amplitude of low-frequency (ALFF), fractional amplitude of low-frequency fluctuation (fALFF), and regional homogeneity (ReHo) [19]. ALFF is the most commonly employed indicator of spontaneous activity at the level of a single voxel, which has high sensitivity and specificity [20]. The fractional amplitude of low-frequency fluctuation (fALFF) is an improved measure of ALFF that allows better control of physiological noise [21]. The ReHo assesses the correlation between the time series of BOLD signals of a given voxel and those of its nearest neighbors at a larger cluster level, thereby providing valuable insights into the regional activity synchronization in the brain [22]. ALFF and fALFF reflect the spontaneous brain activities of a single voxel, and ReHo detects the synchronization in the cluster [4]. These metrics have commonalities and differences, providing complementary information to promote the understanding of regional spontaneous brain activities. Thus, the combination of these three methods may provide more information about the pathophysiological framework of the human brain [23], leading to a better exploration of the structural brain abnormalities in T2DM. Li et al. [4] has already identified concordant regional brain activity abnormalities in patients with T2DM through the meta-analysis combined ALFF, fALFF, and ReHo study.

Rs-fMRI indirectly reflects neural activity by observing changes in blood oxygen levels in the brain [24]. However, abnormal brain function is often closely associated with structural damage [25]. Therefore, it is not sufficient to examine only abnormal brain activity in patients with T2DM. In structural neuroimaging, voxel-based morphometry (VBM) is a fully automated processing method that has been applied to examine abnormal grey matter volume (GMV). The importance of the VBM approach is that it is not biased toward one particular structure and gives an even-handed and comprehensive assessment of anatomical differences throughout the brain [26]. VBM employs statistical techniques to identify differences in brain anatomy between groups of subjects, which in turn can be used to infer the presence of atrophy or tissue expansion in individuals with disease [27]. Many studies have examined GMV alterations in patients with T2DM using VBM [28–30]. Meta-analysis of VBM studies in diabetic patients by Moulton et al. [31] and Wu et al. [30] not only confirmed the presence of abnormal GMVs in patients with T2DM in a wide range of brain regions distributed throughout the brain but also associated with the risk of cognitive impairment and even dementia. The integration of local metrics and VBM can provide a comprehensive analysis to examine the spontaneous neural activity and grey matter structural abnormalities of brain regions in patients with T2DM from both functional and structural perspectives.

Previous researchers have conducted some studies on the local neural activity, FC within the DMN, and GMV in patients with T2DM. However, limited by small sample size, clinical heterogeneity, and methodological differences, previous studies have yielded a large number of inconsistent results. Specifically, small sample size can undermine the reliability of neuroscience research [32], which can increase the number of false-positive and false-negative results [33, 34], overestimate or underestimate effect sizes, and even reverse the direction of the effect, ultimately leading to misleading conclusions [35–37]. As for clinical heterogeneity, research has pointed out that disease duration (e.g., < 5 years vs. > 10 years), the presence of comorbid hypertension or dyslipidemia, and treatment regimens can all influence the neural mechanisms of patients with T2DM [17, 38]. Finally, the seed of FC, and whether whole-brain analysis or ROI analysis is used can also affect the final results [39, 40]. Therefore, an increasing number of researchers are adopting meta-analysis, hoping to combine empirical studies with the same or similar methods for large-sample analyses to detect more common and more reproducible brain functional and structural abnormalities in patients with T2DM. Seven previous meta-analyses have examined the local neural activity and FC in patients with T2DM, and found common abnormal neural activity in the lingual gyrus, superior temporal gyrus, superior frontal gyrus, and cerebellum [41–47]. In addition, ten meta-analyses have examined the gray matter structural changes in patients with T2DM and found common gray matter structural abnormalities in the middle temporal gyrus, insula, precuneus, cerebellum, and basal ganglia [29–31, 41, 43, 44, 48–51]. However, reviewing previous meta-analysis studies, we can find that most studies only focus on research using a certain method or a certain modality of MRI, and thus cannot comprehensively reflect the neural mechanism abnormalities of patients with T2DM. To our knowledge, at present, only four studies have included data from multiple modalities for analysis [41, 43, 44, 51], and one of these studies examined changes in gray and white matter structure [51], while another study focused on T2DM-related cognitive dysfunction [43]. Therefore, it is necessary to conduct a meta-analysis of patients with T2DM to comprehensively examine their local neural activity, FC, and gray matter structural changes.

Given the current status quo of inconsistent results of functional and structural alterations in individual studies, in this study, we conducted a coordinate-based meta-analysis (CBMA) of existing studies in local metrics, DMN, and VBM using anisotropic effect-size-based seeded mapping (AES-SDM) to explore consistent results of functional and structural alterations in brain regions of patients with T2DM. In addition to this, we used the activation likelihood estimation (ALE) and multilevel kernel density analysis (MKDA) as validation analyses for additional validation of the AES-SDM results.

## Methods

### Search strategy and literature screening

The protocol for this neuroimaging meta-analysis was registered on PROSPERO International Prospective Register of Systematic Reviews with the registration number “CRD4202454977” (https://www.crd.york.ac.uk/PROSPERO/export_details_pdf.php). We searched comprehensively in PubMed, Web of Science, and Embase datasets for literature up to July 2023, using the combined keywords: (“T2DM” OR “type 2 diabetes mellitus”) AND (“ALFF” OR “amplitude of low-frequency” OR “fALFF” OR “fractional amplitude of low-frequency” OR “ReHo” OR “regional homogeneity” OR “DMN” OR “default mode network” OR “VBM” OR “voxel-based morphometry”).

For literature selection, we used the following inclusion criteria: (1) employed MRI analysis; (2) compared patients with T2DM and HCs and reported three-dimensional Talairach or Montreal Neurologic Institute (MNI) coordinates of the brain regions; (3) written in English; (4) a sample size of more than 10 in a single group [52, 53]; (5) utilized local metrics, seed-based or ICA methods to analyze DMN, grey matter volume/density for VBM; (6) different studies had partially or wholly overlapped samples, only the study with the largest sample size was included. In addition, studies were excluded if: (1) written in non-English; (2) animals research; (3) articles were review, case report, or meeting abstract; (4) non-MRI studies (5) non-T2DM patients; (6) studies were non-whole-brain analysis; (7) studies not reported Talairach or MNI coordinates; (8) non T2DM vs HCs comparison; (9) Methods not used in the current study; (10) only ANOVA results without post-hoc tests results.

### Quality assessment and data extraction

To ensure high data quality, we used a 20-point checklist to assess the quality of the included studies [54, 55]. The checklist mainly includes the assessment of subject quality (e.g., demographic and clinical characteristics) and the study methodology (e.g., image acquisition, method description), the details of the checklist are presented in Supplementary Table S1. All the above process were conducted by two authors (D.H. and M.Q.Z) independently. If there was a disagreement, it would be solved through discussion with a third author (X.J.Z) until a consensus is reached. After quality assessment, important information was extracted and collated from each included study, mainly including the first author, T2DM type, and statistical threshold.

### Voxel-wise meta-analysis by AES-SDM

An anisotropic effect size seed-based *d* mapping (AES-SDM) software package version 5.15 for Windows was used for the quantitative coordinate-based meta-analysis (https://www.sdmproject.com/software/). First, the corresponding text was generated for each study. The content of the texts includes peak coordinates and the corresponding effect size. The Talairach coordinates can be automatically converted to MNI coordinates using the Lancaster transform by the SDM online converter, and if the study does not specify an effect size, we represent the effect size of the peak coordinates as ‘p’ for increased and ‘n’ for decreased [56–58]. In the current study, there were 5 studies required coordinate conversion [59–62], 3 studies reported results using effect sizes [63–65], and 3 studies reported no significant findings [66–68]. Second, the effect size map of each study was reconstructed using an anisotropic Gaussian kernel [69]. Third, based on the effect size map of each study, the mean effect size map of all studies was calculated using a random effects model that accounts for the sample size, intra-dataset variability, and between-dataset heterogeneity [70]. We used the recommended threshold (peak height *Z* > 1, uncorrected voxel *p* < 0.005, and cluster extent > 10 voxels), which is approximate equivalent to a corrected *p*-value < 0.05. This recommended threshold can achieve an optimal balance between and specificity of false positives [57].

### Analyses of sensitivity, heterogeneity, and publication bias

To verify the robustness and reliability of the meta-analysis results, we conducted a systematic jackknife sensitivity analysis. Jackknife sensitivity analyses were performed using the Leave-One-Out Cross-Validation (LOOCV) method to verify the reproducibility of results [57, 71]. The main analyses were repeated n times (n = the total number of studies) and a different study was discarded each time. The results were considered stable and reproducible if significant brain regions obtained after each LOOCV analysis were present in all or most of the previously significant brain regions [72]. In the current analysis, we removed one study each time to test whether the results regions remained significant. Additionally, we carried out a heterogeneity analysis using a random-effects model with *Q* statistics to explore between-study variability in the results (peak height *Z* > 1, voxel *p* < 0.005, and cluster extent > 10 voxels). Furthermore, we performed Egger’s test to quantify publication biases [73]. Publication bias refers to the tendency of a study with statistically significant results to be published more often than results with no significant results [74]. A *p*-value less than 0.05 was considered statistically significant, which could indicate that the result is only published if they find statistically significant results [75].

### Confirmatory analyses

To further test and validate our results, we conducted two analyses to validate the meta-analysis results: one is ALE analysis using GingerALE 3.0.2 software (https://www.brainmap.org/) with an activation likelihood estimation algorithm. The other is MKDA analysis using a multilevel kernel density analysis toolbox [76] in MATLAB2017b.

In the ALE analysis, Foci reported in the Talairach space were converted into MNI space using the GingerALE software [77]. According to the ALE manual, the required information, such as the MNI coordinates, the name of the study, and the sample size of each study, was compiled into a text file. Subsequently, the file was input into GingerALE software, enabling a fully automated analysis. A statistical threshold (uncorrected *p* < 0.005) was used [78, 79].

In the MKDA analysis, before conducting the analyses, relevant information (e.g., coordinates in MNI or Talairach, the name of the study, contrasts, the total sample size of each study, and coordinate system) was extracted from all the studies to create a text file. The Talairach coordinates reported in the included studies were transformed into the MNI space using Lancaster transformation [56]. To generate comparison indicator maps, the peak coordinates from each relevant contrast map were independently convolved with a 10-mm spherical kernel. According to previous meta-analytic studies, this default kernel size is appropriate [80, 81]. Additionally, the number of Monte Carlo simulations was set to 10,000 iterations. Family-wise error rate correction (FWE) was utilized, and a *p*-value of less than 0.05 was considered statistically significant [82].

## Results

### Included studies and sample characteristics

Following strict inclusion and exclusion criteria, we found 52 studies with 58 datasets that met our requirements and consisted of 2160 patients with T2DM and 2124 HCs. Overall, there were 22 local metrics datasets composed of 570 patients with T2DM and 601 HCs [18, 24, 62, 63, 66, 83–98], 18 DMN-FC datasets included 678 patients with T2DM and 705 HCs [10, 11, 17, 28, 61, 64, 68, 86, 87, 90, 99–106], and 18 VBM datasets included 912 patients with T2DM and 818 HCs [7, 28, 59, 60, 65, 67, 93, 107–117]. Noticeably, there were 5 studies both including local metrics and DMN [66, 86, 90, 106, 118], 1 study both including local metrics and VBM [93], and 1 study both including DMN and VBM [28]. Among the 52 studies, 5 required coordinate conversion from Talairach to MNI space [17, 59–62], which was performed using the SDM online converter with the Lancaster transform [119]. Three studies did not report effect sizes [63–65]; for these, we followed the SDM manual recommendations and applied standardized symbolic notation (using “p” to denote increased activity and “n” to indicate decreased activity). Additionally, 3 studies reported null findings [66–68], and 1 study did not report the mean age or standard deviations (SD) of subjects [93]. Figure S1 shows the flowchart for the identification and exclusion of studies. Table S2–S4 provides detailed information on the studies included.

### The results of the voxel-wise meta-analysis

#### The results of the local metrics meta-analysis

**Fig. 1:**
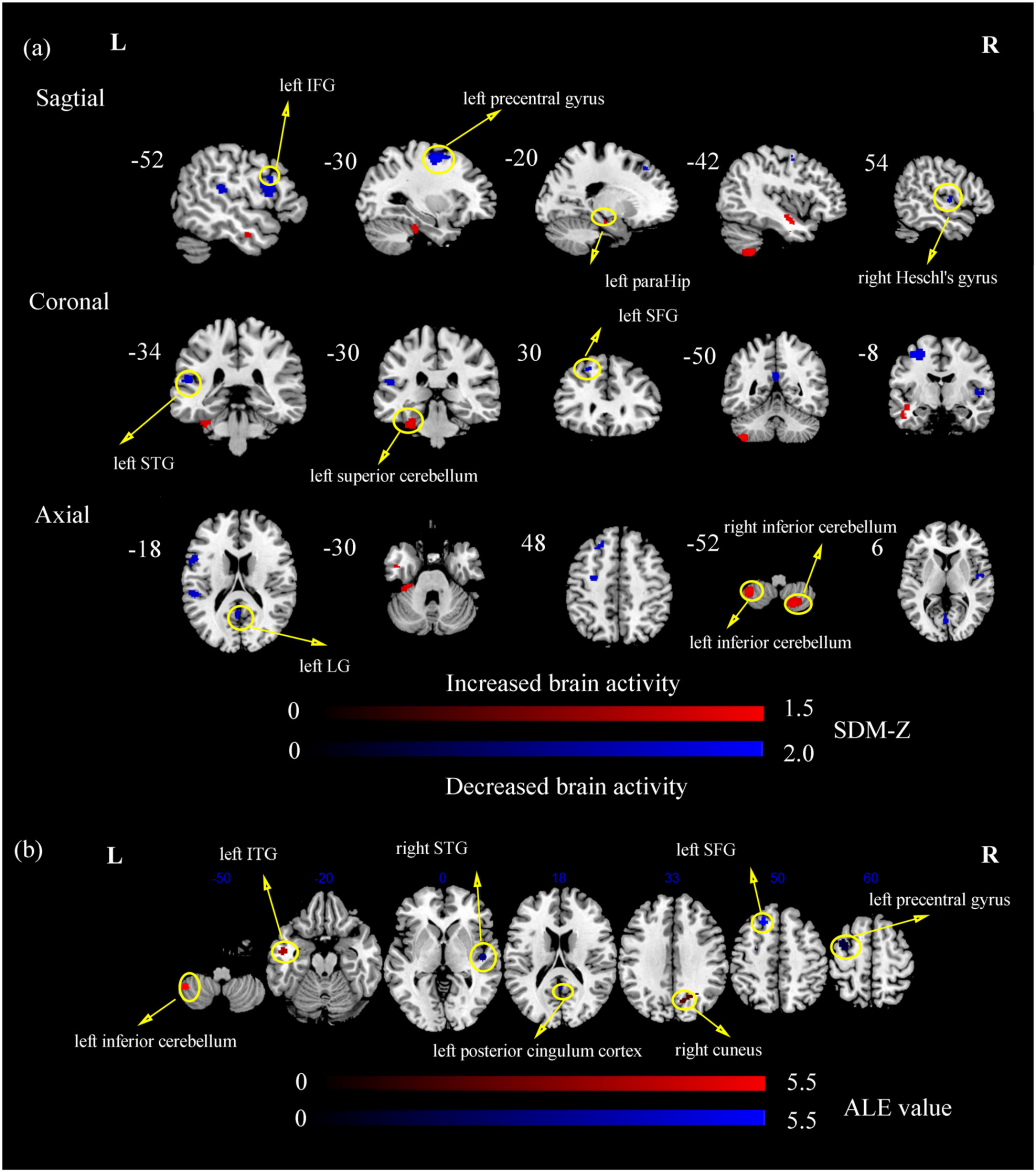
The significant differences in local metrics between patients with T2DM and HCs. **a** The AES-SDM analysis was threshold with an uncorrected p < 0.005. The color bar indicates the maximum and minimum SDM-Z values. Compared with HCs, the increased brain activity in patients with T2DM were marked in red, while the decreased brain activity in patients with T2DM were marked in blue; **b** The ALE analysis was threshold with an uncorrected p-value < 0.005. The color bar indicates the probability of significant ALE values. Compared with HCs, the increased brain activity in patients with T2DM were marked in red, while the decreased brain activity in patients with T2DM were marked in blue. IFG inferior frontal gyrus, paraHip paraHippocampal gyrus, STG superior temporal gyrus, SFG superior frontal gyrus, LG lingual gyrus, ITG inferior temporal gyrus.

Compared to HCs, patients with T2DM showed increased regional brain activity in the bilateral inferior cerebellum, left superior cerebellum, left paraHippocampal gyrus (paraHip), and decreased regional activity in the left precentral gyrus, left lingual gyrus (LG), left inferior frontal gyrus (IFG), left superior temporal gyrus (STG), right Heschl’s gyrus, and left superior frontal gyrus (SFG). The results are presented in Fig. 1 and Table 1.

**Table 1:** Brain regions of consistent abnormal activity in patients with T2DM relative to HCs by AES-SDM, ALE, and MKDA analyses for local metrics.

Brain regions	BA	MNI coordinates			Voxels	*p*-value	Value
		x	y	z			
**AES-SDM**							
Right inferior cerebellum	NA	20	−68	−50	409	<0.005	1.368
Left inferior cerebellum	NA	−42	−50	−52	174	<0.005	1.413
Left superior cerebellum	20	−30	−30	−30	118	<0.005	1.353
Left parahippocampal gyrus	35	−18	−18	−18	13	<0.005	1.213
Left precentral gyrus	6	−30	−6	56	479	<0.005	−1.918
Left lingual gyrus	NA	0	−76	4	287	<0.005	−1.657
Left inferior frontal gyrus	NA	−52	12	28	187	<0.005	−1.642
Left superior temporal gyrus	42	−52	−34	18	70	<0.005	−1.593
Right Heschl’s gyrus	48	54	−8	6	57	<0.005	−1.504
Left superior frontal gyrus	9	−20	30	48	25	<0.005	−1.561
**ALE**							
Right cuneus	7	12	−68	36	148	<0.005	5.065
Left inferior temporal gyrus	20	−50	−8	−24	98	<0.005	5.480
Left inferior cerebellum	NA	−44	−52	−52	89	<0.005	5.171
Left precentral gyrus	6	−34	−2	58	148	<0.005	−5.379
Right superior temporal gyrus	48	48	−16	2	101	<0.005	−4.710
Left posterior cingulum cortex	NA	0	−52	24	91	<0.005	−4.660
Left superior frontal gyrus	NA	−18	28	48	83	<0.005	−4.769
**MKDA**							
None							

The AES-SDM analysis was thresholded with Voxel threshold *p*-value < 0.005; The ALE analysis was thresholded with an uncorrected *p*-value < 0.005.

*BA* Brodmann, *L* left, *R* right, *MNI* montreal neurological institute.

### The results of the DMN meta-analysis

**Fig. 2:**
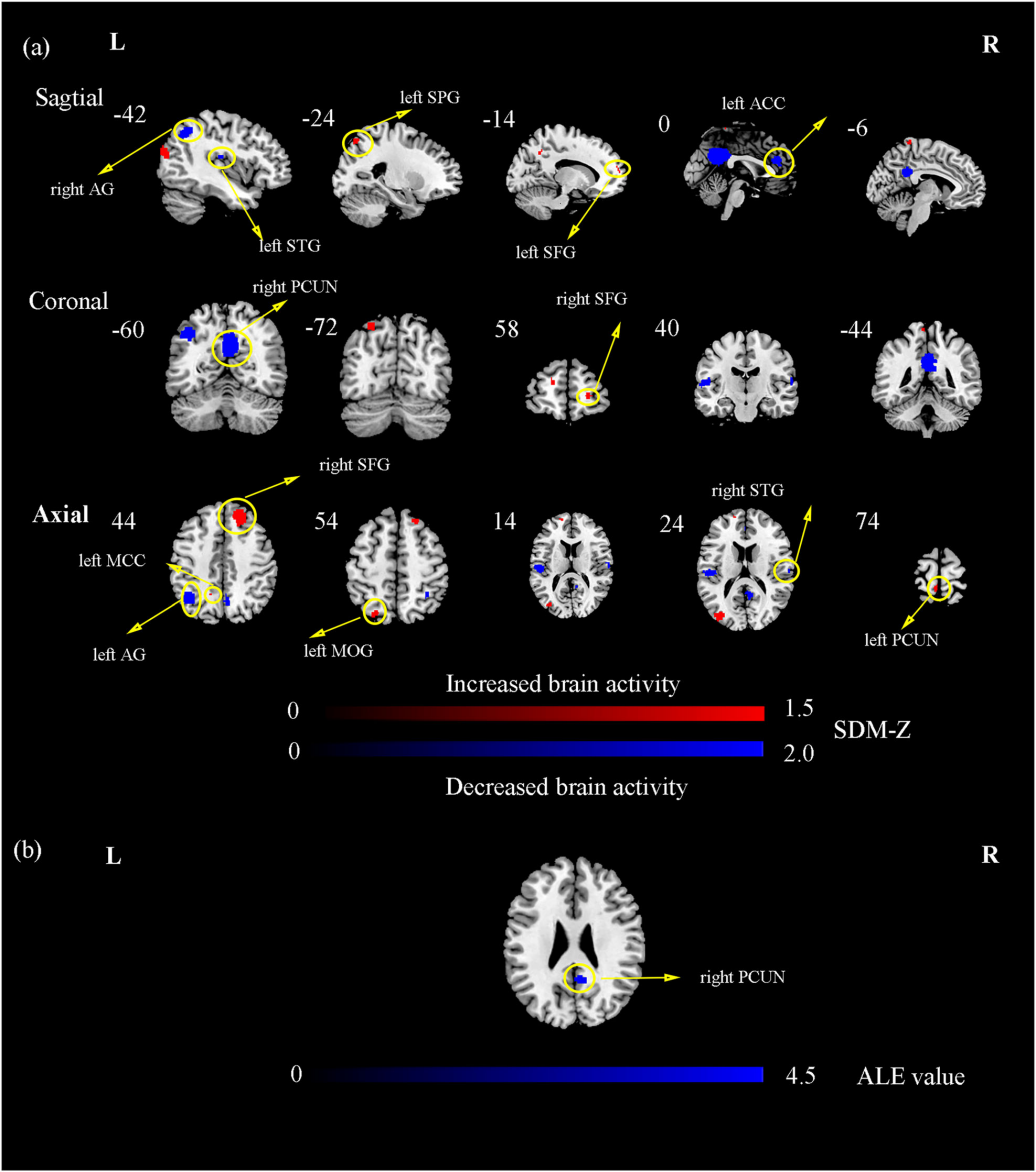
The significant differences in DMN between patients with T2DM and HCs. **a** The AES-SDM analysis was threshold with an uncorrected p-value < 0.005. The color bar indicates the maximum and minimum SDM-Z values. Compared with HCs, the increased FC in patients with T2DM were marked in red, while the decreased FC in Patients with T2DM were marked in blue; **b** The ALE analysis was threshold with an uncorrected p-value < 0.001. The color bar indicates the probability of significant ALE values. Compared with HCs, the decreased FC in patients with T2DM were marked in blue. AG angular gyrus, SPG superior parietal gyrus, ACC anterior cingulate cortex, PCUN precuneus, SFG superior frontal gyrus, MCC middle cingulate cortex, MOG middle occipital gyrus, STG superior temporal gyrus.

Compared to HCs, patients with T2DM showed increased DMN-FC in the right SFG, left middle occipital gyrus (MOG), left superior parietal gyrus (SPG), left precuneus (PCUN), left SFG, left middle cingulate cortex (MCC) and decreased DMN-FC in the right PCUN, bilateral STG, bilateral angular gyrus (AG), and left anterior cingulate cortex (ACC). The results are presented in Fig. 2 and Table 2.

**Table 2:** Brain regions of abnormal DMN-FC in patients with T2DM relative to HCs by AES-SDM, ALE, and MKDA analyses for DMN.

Brain regions	BA	MNI coordinates			Voxels	*p*-value	Value
		x	y	z			
**AES-SDM**							
Right superior frontal gyrus	9	20	36	46	422	<0.005	1.847
Left middle occipital gyrus	19	−34	−78	20	334	<0.005	1.402
Right superior frontal gyrus	11	22	56	2	31	<0.005	1.120
Left superior parietal gyrus	7	−24	−72	54	30	<0.005	1.120
Left precuneus	5	−6	−44	74	24	<0.005	1.070
Left superior frontal gyrus	10	−14	58	14	19	<0.005	1.149
Left middle cingulate cortex	NA	−16	−54	40	12	<0.005	1.205
Right precuneus	23	8	−54	22	1412	<0.005	−2.646
Left superior temporal gyrus	48	−54	−20	14	258	<0.005	−1.756
Left angular gyrus	39	−42	−60	44	218	<0.005	−1.936
Left anterior cingulate cortex	NA	0	40	24	157	<0.005	−1.621
Right angular gyrus	40	36	−54	50	73	<0.005	−1.739
Right superior temporal gyrus	22	62	−14	12	34	<0.005	−1.562
**ALE**							
Right precuneus	31	8	−52	26	76	<0.005	−4.221
**MKDA**							
None							

The AES-SDM analysis was thresholded with Voxel threshold p-value < 0.005; The ALE analysis was thresholded with an uncorrected *p*-value < 0.005.

*BA* Brodmann, *L* left, *R* right, *MNI* montreal neurological institute.

### The results of the VBM meta-analysis

**Fig. 3:**
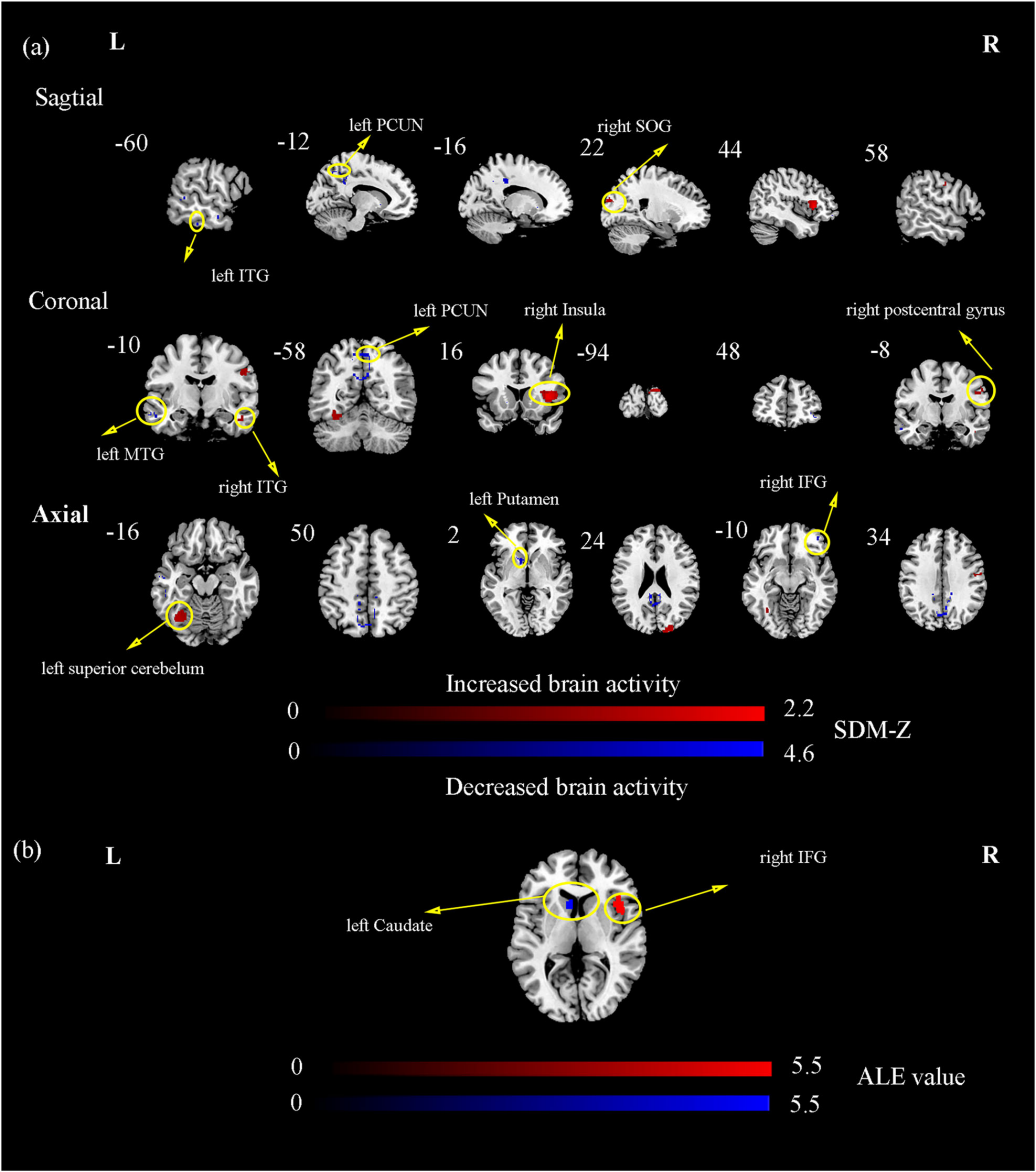
The significant differences in VBM between patients with T2DM and HCs. **a** The AES-SDM analysis was threshold with an uncorrected *p*-value < 0.005. The color bar indicates the maximum and minimum SDM-*Z* values. Compared with HCs, the increased GMV in patients with T2DM were marked in red, while the decreased GMV in patients with T2DM were marked in blue; **b** The ALE analysis was threshold with an uncorrected *p*-value < 0.005. The color bar indicates the probability of significant ALE values. Compared with HCs, the increased GMV in patients with T2DM were marked in red, while the decreased GMV in patients with T2DM were marked in blue. ITG inferior temporal gyrus, PCUN precuneus, SOG superior occipital gyrus, MTG middle temporal gyrus, IFG inferior frontal gyrus.

Compared to HCs, patients with T2DM showed increased GMV in the right insula, left superior cerebellum, right superior occipital gyrus (SOG), right postcentral gyrus, right inferior temporal gyrus (ITG), and decreased GMV in the left PCUN, left ITG, left putamen, right IFG, left middle temporal gyrus (MTG). The results are presented in Fig. 3 and Table 3.

**Table 3:** Brain regions of gray matter structural abnormalities in patients with T2DM relative to HCs by AES-SDM and ALE analyses for VBM.

Brain regions	BA	MNI coordinates			Voxels	*p*-value	Value
		x	y	z			
**AES-SDM**							
Right insula	45	42	20	4	392	<0.005	2.142
Left superior cerebelum	19	−34	−64	−18	158	<0.005	1.506
Right superior occipital gyrus	18	22	−94	24	143	<0.005	1.622
Right postcentral gyrus	3	58	−8	34	50	<0.005	1.382
Right inferior temporal gyrus	20	48	−10	−24	13	<0.005	1.378
Left precuneus	7	−2	−70	46	525	<0.005	−4.524
Left inferior temporal gyrus	20	−56	−26	−24	29	<0.005	−3.690
Left putamen	11	−16	16	−2	21	<0.005	−3.978
Right inferior frontal gyrus	47	44	48	−10	14	<0.005	−3.551
Left precuneus	NA	−12	−58	50	11	<0.005	−3.558
Left middle temporal gyrus	21	−60	−10	−16	10	<0.005	−3.711
Left middle temporal gyrus	22	−58	−42	4	10	<0.005	−3.516
**ALE**							
Right inferior fontal gyrus	48	42	14	6	115	<0.005	5.295
Left caudate	25	−8	16	10	87	<0.005	−5.414
**MKDA**							
None							

The AES-SDM analysis was thresholded with Voxel threshold *p*-value < 0.005; The ALE analysis was thresholded with an uncorrected *p*-value < 0.005.

*BA* Brodmann, *L* left, *R* right, *VBM* voxel-based morphometry, *MNI* montreal neurological institute.

### Analyses of Jackknife sensitivity, heterogeneity, and publication bias

As shown in Tables S5–S7, the Jackknife sensitivity analysis demonstrated high reproducibility in most regions, with all results ensuring a sensitivity of over 77%. In addition, the majority of the results for local metrics showed no heterogeneity, and almost all results for both local metrics and DMN-FC exhibited no publication bias.

### The reproducible meta-analysis results of ALE and MKDA

To test and verify the present results, we conducted two additional meta-analyses (ALE analysis and MKDA analysis). In the results of the local metrics meta-analysis by ALE, we found that patients with T2DM had increased neural activity in the right cuneus, left ITG, left inferior cerebellum, and decreased activity in left precentral gyrus, right STG, left posterior cingulate cortex, and left SFG. In the results of the DMN-FC meta-analysis by ALE, patients with T2DM had decreased FC in right PCUN. In the results of the VBM meta-analysis by ALE, patients with T2DM had increased GMV in right IFG, and decreased GMV in left caudate. The MKDA analysis had no significant result in three analyses. Detailed information about the results of the ALE analysis was provided in Tables 1–3, Figs. 1–3.

## Discussion

In the current study, we used the quantitative AES-SDM meta-analysis to reveal abnormal functional activity and structure in patients with T2DM. Results exhibit altered neural activity and GMV in sensorimotor, cognition and attention related brain regions. In particular, brain regions involved in visual processing, including visual input, visual memory, and visual cognition, showed abnormalities in whatever local neural activity, FC of the DMN, and GMV. To validate the results, other two meta-analyses methods (ALE and MKDA) were employed to corroborate the findings of AES-SDM, and the results of ALE and MKDA were found to be largely consistent with those of AES-SDM.

### Results of local metrics

The current study demonstrated decreased spontaneous neural activity in sensorimotor-related brain regions such as left LG, right Heschl’s gyrus, left STG, and left precentral gyrus in patients with T2DM. Among them, LG is closely related to visual processing, previous study has shown that abnormal brain activity in occipital lobe of patients with T2DM is associated with impaired visual processing speed and spatial memory, potentially affecting global cognitive performance [120]. A study by Peng et al., [121] found that the ReHo value of LG was associated with the Trail-Making Test, which assesses attention, psychomotor speed, and executive function. Previous meta-analysis also found decreased activity in LG [42, 43, 45]. Clinically, patients with T2DM are often associated with retinopathy, which suggests that impaired spontaneous neural activity in the occipital lobe may be associated with retinopathy in patients with T2DM and be present before the onset of symptoms [105]. Although the ALE results did not detect abnormal activity in the LG, but abnormal neural activity was found in an important visual-related brain region (cuneus). This indirectly confirms that there is abnormal activity in the visual-related brain regions of patients with T2DM. The right Heschl’s gyrus includes the primary auditory cortex and lies on the upper surface of the STG [122]. Current studies have shown that the activity of the STG and Heschl’s gyrus is reduced in patients with T2DM. Two meta-analyses of T2DM supported the current results, they both found decreased activity in STG [41, 42]. Another meta-analysis found patients with T2DM had decreased GMV in STG [51]. Other empirical study has also indicated the abnormal degree centrality and FC of the STG and Heschl’s gyrus in early-onset patients with T2DM [28], and another study demonstrated that the metabolic interaction between the IFG and Heschl’s gyrus was a risk factor for T2DM [123]. In addition, previous study have shown that STG is critical for speech perception [124]. Defects in the STG, which is one of the cortical components that serve language processing, may be associated with impaired language processing due to chronic abnormalities in glucose metabolism [125]. As for precentral gyrus, it not only serves as a core sensorimotor brain area but is also closely related to cognitive functions such as executive and attentional functions [126, 127]. A 5-year longitudinal follow-up study, consistent with the current study found that regional neurovascular coupling (NVC) was reduced in precentral gyrus of patients with T2DM, and suggested that reduced NVC in precentral gyrus may be associated with diabetic peripheral neuropathy such as numbness, pain, and other paresthesia [104]. In addition, ALE analysis also identified decreased brain activity in precentral gyrus and STG, which further confirmed the important role of precentral gyrus and STG in T2DM.

The frontal lobe plays a central role in cognition and is associated with emotion, social behavior, motivation, perception, and other processes. Moreover, cognitive functions such as attention, working memory, and decision-making are also commonly attributed to the prefrontal cortex [128]. Current studies have shown that there is decreased activity in the left IFG and the left SFG in patients with T2DM. The decreased activity in SFG was also found in ALE. This is consistent with previous studies, a previous meta-analysis also found decreased activity in the SFG, other 2 meta-analyses of GMV changes in patients with T2DM also found patients with T2DM and T2DM-related cognitive dysfunction (T2DM-CD) both had decreased GMV in the SFG [42, 51]. Other empirical studies have also found functional and structural impairments in the inferior and superior frontal gyrus in patients with T2DM, which are related to cognitive impairments in patients with T2DM [129–131]. The paraHip is an important component of the limbic system and is also associated with emotions, behavior, motivation, and long-term memory [132]. A previous meta-analysis found decreased GMV of the paraHip in patients with T2DM [29]. Another study also indicated that the changes in gray matter density and glucose metabolism in the paraHip of patients with T2DM were related to poorer executive function and memory [133]. In the current study, we found increased activity in the left paraHip of patients with T2DM. However, previous study has shown decreased ALFF in the paraHip of patients with T2DM [96], which may be related to the fact that the patients with T2DM had mild cognitive impairment comorbidity in this study. In addition, consistent with the results of previous meta-analyses [41, 42, 44, 46], AES-SDM and ALE both demonstrated increased neural activity in the cerebellum. The cerebellum is a multifunctional brain region, and previous studies have shown that it is not only closely related to sensorimotor functions [89], but is also extensively involved in processing such as attention, working memory, and social cognition [134].

### Results of DMN

Retinopathy, one of the common complications of diabetes, can lead to visual impairment. Most studies on patients with T2DM with optic nerve-related complications have reported negative changes in the MOG, including reduced dynamic brain activity [1], reduced centrality [135], and reduced FC [86, 125, 136]. Two meta-analyses also reported decreased activity and cerebral blood flow in the MOG of patients with T2DM [46, 137]. However, we found that the increased FC in the left MOG (BA 19) in the current study. The differences from previous studies may be related to different functional subregions of MOG. Previous studies have shown that BA 19 is the visual association cortex responsible for high-level processing and interpretation of visual input [138], and a part of MOG also belongs to BA 17, which is a primary visual cortex and located around the talonavicular fissure in the occipital lobe. It is a typical Koniocortex Cortex that receives inputs from the thalamus lateral geniculate body related to visual information input from the lateral geniculate body of the thalamus [139]. Thus, the different FC of MOG.L in patients with T2DM between the current results and previous studies may reflect subregions where MOG is responsible for different visual functions.

SPG is involved in attention and visuospatial orienting, including information manipulation in working memory [29]. Previous studies have shown that decreased FC of SPG in patients with T2DM is associated with impaired visual conceptual and visuo-motor tracking [140]. Another study also indicated that both short-range and long-range FC strength of the SPG were reduced in patients with T2DM [141]. The abnormal FC of the SPG implied that the neural circuits related to attention and spatial orientation may be impaired in patients with T2DM, which was manifested as inattention and the need for more time to complete spatial orientation function. The ACC is another brain region that is closely related to executive and attention functions. Studies have shown that the ACC plays an important role in the top-down control network, monitoring and resolving conflicts by enhancing the salience of attentional targets and inhibiting distracting stimuli [142, 143]. Previous meta-analysis and empirical studies have indicated that patients with T2DM exhibit increased activity in the ACC, and this hyperactivity is thought to be a compensatory mechanism for cognitive impairment and the maintenance of normal cognition [15, 43, 87, 144]. The current study showed that patients with T2DM had decreased FC in the left ACC, which may reflect that despite compensation in local activity, the overall coordination of the attention network is still impaired, thereby affecting the attention regulation function in patients with T2DM. Previous meta-analysis also found that patients with T2DM had decreased FC between DMN and ACC [47]. The MCC is connected to the ACC and is sometimes referred to as the dorsal ACC, which can integrate different types of information over time to achieve diversity in behavioral associations through the integration of information from various input types [145]. Previous study on the MCC in T2DM has been relatively limited, with only two studies showing increased FC [146] and decreased cortical thickness [147] of the MCC in patients with T2DM, respectively. Moreover, the decreased in MCC cortical thickness was found to be associated with the duration of T2DM. The current study also found increased FC of the MCC in patients with T2DM, which suggests that the MCC may be of great significance in understanding the pathologic mechanisms of T2DM. However, further research is needed to clarify its specific role in T2DM.

The current study showed that there was abnormal FC in the bilateral PCUN and bilateral AG. The current ALE results and previous meta-analysis also found abnormal FC in the right PCUN [47]. The PCUN and AG are core regions of the DMN, which is widely involved in cognitive processing, including self-reference, social cognition, episodic memory, language and semantic memory, and mind wandering [148]. Numerous previous studies have shown that the abnormal FC of the AG and PCUN in patients with T2DM is associated with impairments in executive function and memory performance [140, 149–151]. The current study found that the FC of the left PCUN increased, while that of the right PCUN decreased, which may be associated with hemispheric lateralization. Future studies could further examine the PCUN in terms of hemispheric dominance. In addition, the current study found decreased FC in the STG and increased FC in SFG. Combined the results that we found decreased activity in STG and increased activity in SFG at meta-analysis of local metrics, we speculated that the abnormal FC of STG and SFG were affected by the activity of STG and SFG.

### Results of VBM

In the current findings, significant changes in GMV of sensorimotor-related brain regions, such as postcentral gyrus, PUT.R, and SOG.R, were observed in patients with T2DM. Among them, the postcentral gyrus is a higher-level sensory receptive area, primarily receiving sensory afferents from thalamocortical projections and being responsible for somatosensory processing [152]. Previous meta-analysis and empirical studies have demonstrated that there are abnormalities in the resting-state activity [24, 45], FC [153], effective connectivity [154], topological structure [155], cortical thickness [147, 156], neurovascular coupling of the postcentral gyrus [157], as well as the neural fibers connecting the motor and somatosensory areas in patients with T2DM [158]. Moreover, some studies have pointed out that the abnormalities in the postcentral gyrus of patients with T2DM may be associated with the decreased pain and temperature sensations in diabetic limbs [121]. In addition, previous research has shown that approximately 60% of patients with T2DM will exhibit peripheral neuropathy to varying degrees [159], which may also be related to the damage to the sensorimotor brain regions in patients with T2DM. As an important region of the basal ganglia, putamen is related to motor control, and previous meta-analysis [44] and empirical studies have shown that GMV abnormalities in putamen in patients with T2DM may be associated with impaired motor control [4, 160]. ALE also observed reduced GMV in caudate, which is another important region of basal ganglia. SOG is a core brain region for visual function [161], the current study found that the local spontaneous neural activity, FC of DMN, and grey matter structure showed abnormalities in visual-related regions. This suggested that abnormal visual function may be a core symptom of patients with T2DM.

In the current findings, patients with T2DM also showed significant changes in GMV in bilateral ITG and MTG.L. In previous meta-analysis studies, they only found patients with T2DM had decreased GMV in MTG [29, 30], but there were some studies demonstrated that ITG and MTG had abnormal neural activity and FC [41, 45, 46, 136, 162]. The ITG is involved in cognitive processes such as semantic processing and concept retrieval [163], and contributes to social cognition [164], self-referential processing [165], and emotional stimulus processing [166]. Regarding the temporal lobe in diabetic patients, there is a finding that shows that microscopic abnormalities based on the temporal lobe reveal lower emotional memory performance in diabetic patients [167]. Previous study has linked structural features of the ITG to clinical symptoms, and their results suggested that the GMV content of the ITG may contribute to larger network-level abnormalities associated with negative emotions and rumination [168]. Previous VBM studies have also found that MTG in patients with T2DM exhibit significant alterations in GMV, which have been correlated with poor neurocognitive performance, and the severity of persistent hyperglycemia [45, 110].

INS serves as important components of the limbic system, supporting a variety of functions such as emotion, behavior, motivation, and long-term memory [132, 169]. Cui et al. [24] found that blood glucose variability at different time scales affected grey matter content within the limbic system, with subjects with greater blood glucose variability exhibiting less grey matter and poorer cognitive performance. Previous studies have shown changes in GMV in the bilateral INS [29, 170]. Hirabayashi et al. [171] found that patients with diabetic had significantly lower mean GMV values in the INS than non-diabetic patients. Previous meta-analysis also demonstrated that patients with T2DM had decreased GMV in left insula [29]. However, inconsistent with previous study, the current study demonstrated increased GMV in right insula. On the one hand, this may be influenced by hemispheric lateralization. On the other hand, compared with the previous meta-analysis (7 studies, 8 datasets), the current study included a larger number of studies (16 studies, 18 datasets). Therefore, the results of the two meta-analyses were inconsistent. Future research needs to control for more extraneous variables and include more high-quality studies to further verify the GMV changes in the insula of patients with T2DM. In addition, the current study demonstrated that patients with T2DM had decreased GMV in IFG and PCUN, and had increased GMV in cerebellum. The abnormal GMV in IFG was also examined by ALE, but it found increased GMV in IFG. This may be because the abnormal IFG detected by AES-SDM was located in the orbital part, while it detected by ALE was located in the opercular part. In the previous meta-analysis sections on local metrics and DMN-FC, we similarly found abnormalities in local activity or FC in these brain regions, which suggested that patients with T2DM have both functional and structural alterations in these areas. Consistent with the current study, previous meta-analyses also found abnormalities in the FC and GMV of the PCUN in patients with T2DM [29, 47]. Future studies can further investigate the temporal sequence of the emergence of functional and structural abnormalities in these brain regions.

This study comprehensively explored the changes of local spontaneous neural activity, DMN-FC, and GMV in patients with T2DM. Through integrating three common meta-analysis methods to examine more reliable and reproducible neural markers, thereby deepening the understanding of the neural mechanisms of T2DM. On the one hand, the research results indicated that these neural changes revealed unique patterns of brain function and structure in patients with T2DM, providing potential targets for future clinical interventions, especially in the application of neuromodulation techniques such as transcranial magnetic stimulation (TMS) [172, 173]. Previous studies have shown that TMS treatment targeting specific brain regions identified through meta-analysis can significantly improve cognitive function [174, 175], suggesting that these neural markers have the potential to become new strategies for the intervention of diabetes-related cognitive impairment. On the other hand, from the perspective of the integration of machine learning and MRI, this study provides an important reference for future interdisciplinary exploration. In recent years, with the increasing integration of machine learning and neuroimaging techniques, more and more studies have begun to focus on identifying potential neural markers of T2DM to play a greater role in clinical diagnosis and treatment outcome prediction [176]. The abnormal brain regions were identified through meta-analysis could be used as regions of interest (ROIs) in future machine learning research. Based on these findings, future machine learning research can avoid extensive analysis across the whole brain and instead focus on these highly relevant and influential specific regions, thereby effectively reducing computational burden and enhancing research efficiency. Moreover, focusing on these key regions can also enhance the precision and specificity of the research, providing more reliable and effective input data for machine learning models, which is conducive to improving the accuracy of disease prediction and clinical intervention [177]. In summary, this study not only delves deeply into the neural mechanisms of T2DM at the theoretical level, revealing new perspectives on the disease’s underlying pathology, but also has significant clinical application value. The research results provide important insights for the clinical diagnosis, treatment intervention, and design of personalized medical plans for diabetes. At the same time, based on the ROIs determined in this study, it lays the foundation for future innovative research combining machine learning, which is conducive to promoting the precision and individualization of diabetes treatment and further improving the effectiveness of clinical intervention.

### Limitations

The study in question advances the field’s comprehension of the neural mechanisms associated with T2DM, however, some limitations that should be considered. On the one hand, this study utilized a coordinate-based meta-analysis to delineate the most consistent regions of abnormal spontaneous brain activity in patients with T2DM. This approach is valuable in shedding light on the neural mechanisms underlying T2DM. However, coordinate-based meta-analyses may result in a greater loss of information compared to image-based meta-analyses. Therefore, in this study, we supplemented the results by combining multiple coordinate-based meta-analysis methods as much as possible. Considering that image-based meta-analysis can utilize more whole-brain statistical information, we encourage researchers to share the original statistical parametric maps whenever possible. Further studies should use image-based meta-analyses to validate the current results. On the other hand, because it is difficult to collect fMRI data from patients, to include more studies, we set a sample size of at least 10 as one of the inclusion criteria for this meta-analysis. We hope that future studies will conduct a meta-analysis of T2DM based on a larger sample size to validate the results of this study.

## Conclusion

In the current study, we utilized coordinate-based meta-analysis to conduct the changes of local metrics, DMN, and VBM in patients with T2DM. The results of our comprehensive meta-analysis showed that patients with T2DM have abnormal brain activity and structure, mainly affecting areas related to visual, cognition and attention. These findings provide valuable insights into the pathophysiological mechanisms of T2DM.

## Supplementary information

Supplementary Materials

## Data Availability

All data in this meta-analysis used were extracted from the original study. The data that support the findings of this study are available from the corresponding authors upon reasonable request.

## References

[1] Yang L, Xiao A, Li Q-Y, Zhong H-F, Su T, Shi W-Q, et al. Hyperintensities of middle frontal gyrus in patients with diabetic optic neuropathy: a dynamic amplitude of low-frequency fluctuation study. Aging. 2022;14:1336–50. 35120020 10.18632/aging.203877 PMC8876911

[2] Zhou Z, Sun B, Yu D, Zhu C. Gut microbiota: an important player in type 2 diabetes mellitus. Front Cell Infect Microbiol. 2022;12:834485. 35242721 10.3389/fcimb.2022.834485 PMC8886906

[3] Yang Q, Zhou L, Liu C, Liu D, Zhang Y, Li C, et al. Brain iron deposition in type 2 diabetes mellitus with and without mild cognitive impairment-an in vivo susceptibility mapping study. Brain Imaging Behav. 2018;12:1479–87. 29297155 10.1007/s11682-017-9815-7

[4] Li M, Li Y, Zhao K, Tan X, Chen Y, Qin C, et al. Changes in the structure, perfusion, and function of the hippocampus in type 2 diabetes mellitus. Front Neurosci. 2022;16:1070911. 36699515 10.3389/fnins.2022.1070911 PMC9868830

[5] Li W, Sun L, Li G, Xiao S. Prevalence, influence factors and cognitive characteristics of Mild cognitive impairment in type 2 diabetes mellitus. Front Aging Neurosci. 2019;11:180. 31417393 10.3389/fnagi.2019.00180 PMC6682644

[6] Feng M, Zhang Y, Wen Z, Hou X, Ye Y, Fu C, et al. Early fractional amplitude of low frequency fluctuation can predict the efficacy of transcutaneous auricular vagus nerve stimulation treatment for migraine without aura. Front Mol Neurosci. 2022;15:778139. 35283732 10.3389/fnmol.2022.778139 PMC8908103

[7] Zhang Y, Zhang X, Zhang J, Liu C, Yuan Q, Yin X, et al. Gray matter volume abnormalities in type 2 diabetes mellitus with and without mild cognitive impairment. Neurosci Lett. 2014;562:1–6. 24434688 10.1016/j.neulet.2014.01.006

[8] Glerean E, Salmi J, Lahnakoski JM, Jääskeläinen IP, Sams M. Functional magnetic resonance imaging phase synchronization as a measure of dynamic functional connectivity. Brain Connect. 2012;2:91–101. 22559794 10.1089/brain.2011.0068 PMC3624768

[9] Battaglia D, Brovelli A. Functional connectivity and neuronal dynamics: insights from computational methods. The Cognitive Neurosciences, Sixth Edition 2020. pp 739–747.

[10] Cui Y, Jiao Y, Chen H-J, Ding J, Luo B, Peng C-Y, et al. Aberrant functional connectivity of default-mode network in type 2 diabetes patients. Eur Radiol. 2015;25:3238–46. 25903712 10.1007/s00330-015-3746-8 PMC4595523

[11] Xia W, Rao H, Spaeth AM, Huang R, Tian S, Cai R, et al. Blood pressure is associated with cerebral blood flow alterations in patients with T2DM as revealed by perfusion functional MRI. Medicine. 2015;94:e2231. 26632913 10.1097/MD.0000000000002231 PMC4674216

[12] Ishibashi K, Sakurai K, Shimoji K, Tokumaru AM, Ishii K. Altered functional connectivity of the default mode network by glucose loading in young, healthy participants. BMC Neurosci. 2018;19:33. 29855257 10.1186/s12868-018-0433-0 PMC5984391

[13] Marder TJ, Flores VL, Bolo NR, Hoogenboom WS, Simonson DC, Jacobson AM, et al. Task-induced brain activity patterns in type 2 diabetes: a potential biomarker for cognitive decline. Diabetes. 2014;63:3112–9. 24705405 10.2337/db13-1783 PMC4141362

[14] Ito T, Hearne LJ, Cole MW. A cortical hierarchy of localized and distributed processes revealed via dissociation of task activations, connectivity changes, and intrinsic timescales. Neuroimage. 2020;221:117141. 32663642 10.1016/j.neuroimage.2020.117141 PMC7779074

[15] Liu D, Duan S, Zhou C, Wei P, Chen L, Yin X, et al. Altered brain functional hubs and connectivity in type 2 diabetes mellitus patients: a resting-state fMRI study. Front Aging Neurosci. 2018;10:55. 29563869 10.3389/fnagi.2018.00055 PMC5845755

[16] Mantini D, Perrucci MG, Del Gratta C, Romani GL, Corbetta M. Electrophysiological signatures of resting state networks in the human brain. Proc Natl Acad Sci USA. 2007;104:13170–5. 17670949 10.1073/pnas.0700668104 PMC1941820

[17] Musen G, Jacobson AM, Bolo NR, Simonson DC, Shenton ME, McCartney RL, et al. Resting-state brain functional connectivity is altered in type 2 diabetes. Diabetes. 2012;61:2375–9. 22664957 10.2337/db11-1669 PMC3425418

[18] Zhang Q, Zhang P, Yan R, Xu X, Mao C, Liu X, et al. A single-blinded trial using resting-state functional magnetic resonance imaging of brain activity in patients with type 2 diabetes and painful neuropathy. Diabetes Ther. 2019;10:135–47. 30506341 10.1007/s13300-018-0534-x PMC6349288

[19] Yang J, Gohel S, Vachha B. Current methods and new directions in resting state fMRI. Clin Imaging. 2020;65:47–53. 32353718 10.1016/j.clinimag.2020.04.004 PMC7365764

[20] Zang Y-F, He Y, Zhu C-Z, Cao Q-J, Sui M-Q, Liang M, et al. Altered baseline brain activity in children with ADHD revealed by resting-state functional MRI. Brain Dev. 2007;29:83–91. 16919409 10.1016/j.braindev.2006.07.002

[21] Zou Q-H, Zhu C-Z, Yang Y, Zuo X-N, Long X-Y, Cao Q-J, et al. An improved approach to detection of amplitude of low-frequency fluctuation (ALFF) for resting-state fMRI: fractional ALFF. J Neurosci Methods. 2008;172:137–41. 18501969 10.1016/j.jneumeth.2008.04.012 PMC3902859

[22] Zang Y, Jiang T, Lu Y, He Y, Tian L. Regional homogeneity approach to fMRI data analysis. Neuroimage. 2004;22:394–400. 15110032 10.1016/j.neuroimage.2003.12.030

[23] Wang Q, Wang C, Deng Q, Zhan L, Tang Y, Li H, et al. Alterations of regional spontaneous brain activities in anxiety disorders: a meta-analysis. J Affect Disord. 2022;296:233–40. 34619449 10.1016/j.jad.2021.09.062

[24] Cui Y, Jiao Y, Chen Y-C, Wang K, Gao B, Wen S, et al. Altered spontaneous brain activity in type 2 diabetes: a resting-state functional MRI study. Diabetes. 2014;63:749–60. 24353185 10.2337/db13-0519

[25] Park H-J, Friston K. Structural and functional brain networks: from connections to cognition. Science. 2013;342:1238411. 24179229 10.1126/science.1238411

[26] Ashburner J, Friston KJ. Voxel-based morphometry–the methods. Neuroimage. 2000;11:805–21. 10860804 10.1006/nimg.2000.0582

[27] Whitwell JL. Voxel-based morphometry: an automated technique for assessing structural changes in the brain. J Neurosci. 2009;29:9661–4. 19657018 10.1523/JNEUROSCI.2160-09.2009 PMC6666603

[28] Feng Y, Li Y, Tan X, Liang Y, Ma X, Chen Y, et al. Altered gray matter volume, functional connectivity, and degree centrality in early-onset type 2 diabetes mellitus. Front Neurol. 2021;12:697349. 34566841 10.3389/fneur.2021.697349 PMC8459017

[29] Liu J, Liu T, Wang W, Ma L, Ma X, Shi S, et al. Reduced gray matter volume in patients with type 2 diabetes mellitus. Front Aging Neurosci. 2017;9:161. 28588480 10.3389/fnagi.2017.00161 PMC5439076

[30] Wu G, Lin L, Zhang Q, Wu J. Brain gray matter changes in type 2 diabetes mellitus: a meta-analysis of whole-brain voxel-based morphometry study. J Diabetes Complications. 2017;31:1698–703. 29033311 10.1016/j.jdiacomp.2017.09.001

[31] Moulton CD, Costafreda SG, Horton P, Ismail K, Fu CHY. Meta-analyses of structural regional cerebral effects in type 1 and type 2 diabetes. Brain Imaging Behav. 2015;9:651–62. 25563229 10.1007/s11682-014-9348-2

[32] Button KS, Ioannidis JPA, Mokrysz C, Nosek BA, Flint J, Robinson ESJ, et al. Power failure: why small sample size undermines the reliability of neuroscience. Nat Rev Neurosci. 2013;14:365–76. 23571845 10.1038/nrn3475

[33] Chen X, Lu B, Yan C-G. Reproducibility of R-fMRI metrics on the impact of different strategies for multiple comparison correction and sample sizes. Hum Brain Mapp. 2018;39:300–18. 29024299 10.1002/hbm.23843 PMC6866539

[34] Cremers HR, Wager TD, Yarkoni T. The relation between statistical power and inference in fMRI. PLoS One. 2017;12:e0184923. 29155843 10.1371/journal.pone.0184923 PMC5695788

[35] Gelman A, Carlin J. Beyond power calculations: assessing type S (Sign) and type M (Magnitude) errors. Perspect Psychol Sci. 2014;9:641–51. 26186114 10.1177/1745691614551642

[36] Lorca-Puls DL, Gajardo-Vidal A, White J, Seghier ML, Leff AP, Green DW, et al. The impact of sample size on the reproducibility of voxel-based lesion-deficit mappings. Neuropsychologia. 2018;115:101–11. 29550526 10.1016/j.neuropsychologia.2018.03.014 PMC6018568

[37] Nissen SB, Magidson T, Gross K, Bergstrom CT. Publication bias and the canonization of false facts. Elife. 2016;5:e21451. 27995896 10.7554/eLife.21451 PMC5173326

[38] Strachan MWJ, Reynolds RM, Marioni RE, Price JF. Cognitive function, dementia and type 2 diabetes mellitus in the elderly. Nat Rev Endocrinol. 2011;7:108–14. 21263438 10.1038/nrendo.2010.228

[39] Müller VI, Cieslik EC, Laird AR, Fox PT, Radua J, Mataix-Cols D, et al. Ten simple rules for neuroimaging meta-analysis. Neurosci Biobehav Rev. 2018;84:151–61. 29180258 10.1016/j.neubiorev.2017.11.012 PMC5918306

[40] Tahmasian M, Sepehry AA, Samea F, Khodadadifar T, Soltaninejad Z, Javaheripour N, et al. Practical recommendations to conduct a neuroimaging meta-analysis for neuropsychiatric disorders. Hum Brain Mapp. 2019;40:5142–54. 31379049 10.1002/hbm.24746 PMC6865620

[41] Yao L, Yang C, Zhang W, Li S, Li Q, Chen L, et al. A multimodal meta-analysis of regional structural and functional brain alterations in type 2 diabetes. Front Neuroendocrinol. 2021;62:100915. 33862036 10.1016/j.yfrne.2021.100915

[42] Liu J, Li Y, Yang X, Xu H, Ren J, Zhou P. Regional spontaneous neural activity alterations in type 2 diabetes mellitus: a meta-analysis of resting-state functional MRI studies. Front Aging Neurosci. 2021;13:678359. 34220486 10.3389/fnagi.2021.678359 PMC8245688

[43] Dai P, Yu Y, Sun Q, Yang Y, Hu B, Xie H, et al. Abnormal changes of brain function and structure in patients with T2DM-related cognitive impairment: a neuroimaging meta-analysis and an independent validation. Nutr Diabetes. 2024;14:91. 39528442 10.1038/s41387-024-00348-5 PMC11554684

[44] Antal B, McMahon LP, Sultan SF, Lithen A, Wexler DJ, Dickerson B, et al. Type 2 diabetes mellitus accelerates brain aging and cognitive decline: complementary findings from UK Biobank and meta-analyses. Elife. 2022;11:e73138. 35608247 10.7554/eLife.73138 PMC9132576

[45] Xia W, Chen Y-C, Ma J. Resting-state brain anomalies in type 2 diabetes: a meta-analysis. Front Aging Neurosci. 2017;9:14. 28197096 10.3389/fnagi.2017.00014 PMC5281680

[46] Xie H, Yu Y, Yang Y, Sun Q, Li Z-Y, Ni M-H, et al. Commonalities and distinctions between the type 2 diabetes mellitus and Alzheimer’s disease: a systematic review and multimodal neuroimaging meta-analysis. Front Neurosci. 2023;17:1301778. 38125399 10.3389/fnins.2023.1301778 PMC10731270

[47] Meng J, Liu J, Li H, Gao Y, Cao L, He Y, et al. Impairments in intrinsic functional networks in type 2 diabetes: a meta-analysis of resting-state functional connectivity. Front Neuroendocrinol. 2022;66:100992. 35278579 10.1016/j.yfrne.2022.100992

[48] Yu KKK, Cheing GLY, Cheung C, Kranz GS, Cheung AK-K. Gray matter abnormalities in type 1 and type 2 diabetes: a dual disorder ALE quantification. Front Neurosci. 2021;15:638861. 34163319 10.3389/fnins.2021.638861 PMC8215122

[49] Zhang T, Shaw M, Cherbuin N. Association between type 2 diabetes mellitus and brain atrophy: a meta-analysis. Diabetes Metab J. 2022;46:781–802. 35255549 10.4093/dmj.2021.0189 PMC9532183

[50] Hughes TM, Sink KM, Williamson JD, Hugenschmidt CE, Wagner BC, Whitlow CT, et al. Relationships between cerebral structure and cognitive function in African Americans with type 2 diabetes. J Diabetes Complications. 2018;32:916–21. 30042057 10.1016/j.jdiacomp.2018.05.017 PMC6138531

[51] Ma T, Li Z-Y, Yu Y, Hu B, Han Y, Ni M-H, et al. Gray and white matter abnormality in patients with T2DM-related cognitive dysfunction: a systemic review and meta-analysis. Nutr Diabetes. 2022;12:39. 35970833 10.1038/s41387-022-00214-2 PMC9378704

[52] Zhou X, Teng T, Zhang Y, Del Giovane C, Furukawa TA, Weisz JR, et al. Comparative efficacy and acceptability of antidepressants, psychotherapies, and their combination for acute treatment of children and adolescents with depressive disorder: a systematic review and network meta-analysis. Lancet Psychiatry. 2020;7:581–601. 32563306 10.1016/S2215-0366(20)30137-1 PMC7303954

[53] Cipriani A, Zhou X, Del Giovane C, Hetrick SE, Qin B, Whittington C, et al. Comparative efficacy and tolerability of antidepressants for major depressive disorder in children and adolescents: a network meta-analysis. Lancet. 2016;388:881–90. 27289172 10.1016/S0140-6736(16)30385-3

[54] Iwabuchi SJ, Krishnadas R, Li C, Auer DP, Radua J, Palaniyappan L. Localized connectivity in depression: a meta-analysis of resting state functional imaging studies. Neurosci Biobehav Rev. 2015;51:77–86. 25597656 10.1016/j.neubiorev.2015.01.006

[55] Pan P, Zhu L, Yu T, Shi H, Zhang B, Qin R, et al. Aberrant spontaneous low-frequency brain activity in amnestic mild cognitive impairment: a meta-analysis of resting-state fMRI studies. Ageing Res Rev. 2017;35:12–21. 28017880 10.1016/j.arr.2016.12.001

[56] Lancaster JL, Tordesillas-Gutiérrez D, Martinez M, Salinas F, Evans A, Zilles K, et al. Bias between MNI and Talairach coordinates analyzed using the ICBM-152 brain template. Hum Brain Mapp. 2007;28:1194–205. 17266101 10.1002/hbm.20345 PMC6871323

[57] Radua J, Mataix-Cols D, Phillips ML, El-Hage W, Kronhaus DM, Cardoner N, et al. A new meta-analytic method for neuroimaging studies that combines reported peak coordinates and statistical parametric maps. Eur Psychiatry. 2012;27:605–11. 21658917 10.1016/j.eurpsy.2011.04.001

[58] Lim L, Howells H, Radua J, Rubia K. Aberrant structural connectivity in childhood maltreatment: a meta-analysis. Neurosci Biobehav Rev. 2020;116:406–14. 32659288 10.1016/j.neubiorev.2020.07.004

[59] Nouwen A, Chambers A, Chechlacz M, Higgs S, Blissett J, Barrett TG, et al. Microstructural abnormalities in white and gray matter in obese adolescents with and without type 2 diabetes. Neuroimage Clin. 2017;16:43–51. 28752059 10.1016/j.nicl.2017.07.004 PMC5514690

[60] Chen Z, Li L, Sun J, Ma L. Mapping the brain in type II diabetes: voxel-based morphometry using DARTEL. Eur J Radiol. 2012;81:1870–6. 21546180 10.1016/j.ejrad.2011.04.025

[61] Yu Y, Yan L-F, Sun Q, Hu B, Zhang J, Yang Y, et al. Neurovascular decoupling in type 2 diabetes mellitus without mild cognitive impairment: potential biomarker for early cognitive impairment. Neuroimage. 2019;200:644–58. 31252056 10.1016/j.neuroimage.2019.06.058

[62] Xin H, Fu Y, Feng M, Wang S, Sui C, Gao Y, et al. Altered intrinsic brain activity related to neurologic and motor dysfunction in diabetic peripheral neuropathy patients. J Clin Endocrinol Metab. 2023;108:802–11. 36333998 10.1210/clinem/dgac651

[63] Qian H, Qin D, Qi S, Teng Y, Li C, Yao Y, et al. Less is better: single-digit brain functional connections predict T2DM and T2DM-induced cognitive impairment. Front Neurosci. 2020;14:588684. 33505236 10.3389/fnins.2020.588684 PMC7829678

[64] Hoogenboom WS, Marder TJ, Flores VL, Huisman S, Eaton HP, Schneiderman JS, et al. Cerebral white matter integrity and resting-state functional connectivity in middle-aged patients with type 2 diabetes. Diabetes. 2014;63:728–38. 24203723 10.2337/db13-1219 PMC3900542

[65] Crisóstomo J, Duarte JV, Moreno C, Gomes L, Castelo-Branco M. A novel morphometric signature of brain alterations in type 2 diabetes: patterns of changed cortical gyrification. Eur J Neurosci. 2021;54:6322–33. 34390585 10.1111/ejn.15424 PMC9291170

[66] Li M, Li Y, Zhao K, Qin C, Chen Y, Liu Y, et al. Abnormal cerebral blood flow and brain function in type 2 diabetes mellitus. Endocrine. 2024;85:433–42. 37340286 10.1007/s12020-023-03342-6

[67] Chen J, Zhang J, Liu X, Wang X, Xu X, Li H, et al. Abnormal subcortical nuclei shapes in patients with type 2 diabetes mellitus. Eur Radiol. 2017;27:4247–56. 28374074 10.1007/s00330-017-4790-3

[68] Lips MA, Wijngaarden MA, van der Grond J, van Buchem MA, de Groot GH, Rombouts SARB, et al. Resting-state functional connectivity of brain regions involved in cognitive control, motivation, and reward is enhanced in obese females. Am J Clin Nutr. 2014;100:524–31. 24965310 10.3945/ajcn.113.080671

[69] Radua J, Rubia K, Canales-Rodríguez EJ, Pomarol-Clotet E, Fusar-Poli P, Mataix-Cols D. Anisotropic kernels for coordinate-based meta-analyses of neuroimaging studies. Front Psychiatry. 2014;5:13. 24575054 10.3389/fpsyt.2014.00013 PMC3919071

[70] Radua J, Mataix-Cols D. Meta-analytic methods for neuroimaging data explained. Biol Mood Anxiety Disord. 2012;2:6. 22737993 10.1186/2045-5380-2-6 PMC3384225

[71] Radua J, Mataix-Cols D. Voxel-wise meta-analysis of grey matter changes in obsessive-compulsive disorder. Br J Psychiatry. 2009;195:393–402. 19880927 10.1192/bjp.bp.108.055046

[72] Duko B, Ayano G, Pereira G, Betts K, Alati R. Prenatal tobacco use and the risk of mood disorders in offspring: a systematic review and meta-analysis. Soc Psychiatry Psychiatr Epidemiol. 2020;55:1549–62. 32895729 10.1007/s00127-020-01949-y

[73] Egger M, Davey Smith G, Schneider M, Minder C. Bias in meta-analysis detected by a simple, graphical test. BMJ. 1997;315:629–34. 9310563 10.1136/bmj.315.7109.629 PMC2127453

[74] Sterne JAC, Sutton AJ, Ioannidis JPA, Terrin N, Jones DR, Lau J, et al. Recommendations for examining and interpreting funnel plot asymmetry in meta-analyses of randomised controlled trials. BMJ. 2011;343:d4002. 21784880 10.1136/bmj.d4002

[75] Zhen D, Xia W, Yi ZQ, Zhao PW, Zhong JG, Shi HC, et al. Alterations of brain local functional connectivity in amnestic mild cognitive impairment. Transl Neurodegener. 2018;7:26. 30443345 10.1186/s40035-018-0134-8 PMC6220503

[76] Wager TD, Lindquist M, Kaplan L. Meta-analysis of functional neuroimaging data: current and future directions. Soc Cogn Affect Neurosci. 2007;2:150–8. 18985131 10.1093/scan/nsm015 PMC2555451

[77] Xia R, Ren J, Wang M, Wan Y, Dai Y, Li X, et al. Effect of acupuncture on brain functional networks in patients with mild cognitive impairment: an activation likelihood estimation meta-analysis. Acupunct Med. 2023;41:259–67. 36790017 10.1177/09645284221146199

[78] Andrzejewski JA, Greenberg T, Carlson JM. Neural correlates of aversive anticipation: an activation likelihood estimate meta-analysis across multiple sensory modalities. Cogn Affect Behav Neurosci. 2019;19:1379–90. 31502205 10.3758/s13415-019-00747-7

[79] Gavazzi G, Giovannelli F, Currò T, Mascalchi M, Viggiano MP. Contiguity of proactive and reactive inhibitory brain areas: a cognitive model based on ALE meta-analyses. Brain Imaging Behav. 2021;15:2199–214. 32748318 10.1007/s11682-020-00369-5 PMC8413163

[80] Salimi-Khorshidi G, Smith SM, Keltner JR, Wager TD, Nichols TE. Meta-analysis of neuroimaging data: a comparison of image-based and coordinate-based pooling of studies. Neuroimage. 2009;45:810–23. 19166944 10.1016/j.neuroimage.2008.12.039

[81] Wager TD, Smith EE. Neuroimaging studies of working memory: a meta-analysis. Cogn Affect Behav Neurosci. 2003;3:255–74. 15040547 10.3758/cabn.3.4.255

[82] Ikutani Y, Itoh TD, Kubo T. The neural bases of program comprehension: a coordinate-based fMRI meta-analysis. bioRxiv 2021;2021.04.15.439937.

[83] Bao Y-W, Shea Y-F, Chiu PK-C, Kwan JSK, Chan FH-W, Chow W-S, et al. The fractional amplitude of low-frequency fluctuations signals related to amyloid uptake in high-risk populations-a pilot fMRI study. Front Aging Neurosci. 2022;14:956222. 35966783 10.3389/fnagi.2022.956222 PMC9372772

[84] Chen J, Huang X, Tang Q, Xiang Z, Xu Y, Liu T, et al. Altered regional homogeneity in patients with diabetic erectile dysfunction: a resting-state fMRI study. Front Endocrinol. 2022;13:817523. 10.3389/fendo.2022.817523 PMC9355575 35937825

[85] Li C, Jin R, Liu K, Li Y, Zuo Z, Tong H, et al. White matter atrophy in type 2 diabetes mellitus patients with mild cognitive impairment. Front Neurosci. 2020;14:602501. 33536867 10.3389/fnins.2020.602501 PMC7848149

[86] Liu D, Duan S, Wei P, Chen L, Wang J, Zhang J. Aberrant brain spontaneous activity and synchronization in type 2 diabetes mellitus patients: a resting-state functional MRI study. Front Aging Neurosci. 2020;12:181. 32612525 10.3389/fnagi.2020.00181 PMC7308457

[87] Liu D, Duan S, Zhang J, Zhou C, Liang M, Yin X, et al. Aberrant brain regional homogeneity and functional connectivity in middle-aged T2DM patients: a resting-state functional MRI study. Front Hum Neurosci. 2016;10:490. 27729856 10.3389/fnhum.2016.00490 PMC5037166

[88] Liu M, Li J, Li J, Yang H, Yao Q, Zheng X, et al. Altered spontaneous brain activity in patients with diabetic osteoporosis using regional homogeneity: a resting-state functional magnetic resonance imaging study. Front Aging Neurosci. 2022;14:851929. 35601621 10.3389/fnagi.2022.851929 PMC9120436

[89] Prati JM, Pontes-Silva A, Gianlorenço ACL. The cerebellum and its connections to other brain structures involved in motor and non-motor functions: a comprehensive review. Behav Brain Res. 2024;465:114933. 38458437 10.1016/j.bbr.2024.114933

[90] Qin D, Qian H, Qi S, Teng Y, Wu J. Analysis of RS-FMRI images clarifies brain alterations in type 2 diabetes mellitus patients with cognitive impairment. J Mech Med Biol. 2021;21:2140015.

[91] Shi W-Q, Tang L-Y, Lin Q, Li B, Jiang N, Zhu P-W, et al. Altered spontaneous brain activity patterns in diabetic patients with vitreous hemorrhage using amplitude of low‑frequency fluctuation: a resting‑state fMRI study. Mol Med Rep. 2020;22:2291–9. 32705185 10.3892/mmr.2020.11294 PMC7411342

[92] Wang C-X, Fu K-L, Liu H-J, Xing F, Zhang S-Y. Spontaneous brain activity in type 2 diabetics revealed by amplitude of low-frequency fluctuations and its association with diabetic vascular disease: a resting-state FMRI study. PLoS One. 2014;9:e108883. 25272033 10.1371/journal.pone.0108883 PMC4182760

[93] Wang YF, Kong X, Lu GM, Zhang LJ. Diabetes mellitus is associated with more severe brain spontaneous activity impairment and gray matter loss in patients with cirrhosis. Sci Rep. 2017;7:7775. 28798299 10.1038/s41598-017-08075-x PMC5552886

[94] Xia W, Wang S, Sun Z, Bai F, Zhou Y, Yang Y, et al. Altered baseline brain activity in type 2 diabetes: a resting-state fMRI study. Psychoneuroendocrinology. 2013;38:2493–501. 23786881 10.1016/j.psyneuen.2013.05.012

[95] Xiong Y, Chen X, Zhao X, Fan Y, Zhang Q, Zhu W. Altered regional homogeneity and functional brain networks in type 2 diabetes with and without mild cognitive impairment. Sci Rep. 2020;10:21254. 33277510 10.1038/s41598-020-76495-3 PMC7718881

[96] Zhou X, Zhang J, Chen Y, Ma T, Wang Y, Wang J, et al. Aggravated cognitive and brain functional impairment in mild cognitive impairment patients with type 2 diabetes: a resting-state functional MRI study. J Alzheimers Dis. 2014;41:925–35. 24705547 10.3233/JAD-132354

[97] Xiang Z, Huang Y, Xu Y, Liu X, Huang X, Liu T, et al. Altered brain activity in diabetic patients with erectile dysfunction revealed by fractional amplitude of low-frequency fluctuation: a resting-state fMRI study. Andrology. 2024;12:68–74. 37058742 10.1111/andr.13442

[98] Wang Z-L, Zou L, Lu Z-W, Xie X-Q, Jia Z-Z, Pan C-J, et al. Abnormal spontaneous brain activity in type 2 diabetic retinopathy revealed by amplitude of low-frequency fluctuations: a resting-state fMRI study. Clin Radiol. 2017;72:340.e1–340.e7. 28041652 10.1016/j.crad.2016.11.012

[99] Huang Y, Zhang D, Zhang X, Cheng M, Yang Z, Gao J, et al. Altered functional hubs and connectivity in type 2 diabetes mellitus with and without mild cognitive impairment. Front Neurol. 2022;13:1062816. 36578308 10.3389/fneur.2022.1062816 PMC9792165

[100] Lei Y, Zhang D, Qi F, Gao J, Tang M, Ai K, et al. Dysfunctional interaction between the dorsal attention network and the default mode network in patients with type 2 diabetes mellitus. Front Hum Neurosci. 2021;15:796386. 35002661 10.3389/fnhum.2021.796386 PMC8741406

[101] Li C, Zuo Z, Liu D, Jiang R, Li Y, Li H, et al. Type 2 diabetes mellitus may exacerbate gray matter atrophy in patients with early-onset mild cognitive impairment. Front Neurosci. 2020;14:856. 32848591 10.3389/fnins.2020.00856 PMC7432296

[102] Wang M, Zhang D, Gao J, Qi F, Su Y, Lei Y, et al. Abnormal functional connectivity in the right dorsal anterior insula associated with cognitive dysfunction in patients with type 2 diabetes mellitus. Brain Behav. 2022;12:e2553. 35543304 10.1002/brb3.2553 PMC9226846

[103] Wu J, Kang S, Su J, Liu K, Fan L, Ma X, et al. Altered functional network connectivity of precuneus and executive control networks in type 2 diabetes mellitus without cognitive impairment. Front Neurosci. 2022;16:887713. 35833084 10.3389/fnins.2022.887713 PMC9271612

[104] Xia W, Luo Y, Chen Y-C, Chen H, Ma J, Yin X. Glucose fluctuations are linked to disrupted brain functional architecture and cognitive impairment. J Alzheimers Dis. 2020;74:603–13. 32065795 10.3233/JAD-191217

[105] Zhang Y, Wang J, Wei P, Zhang J, Zhang G, Pan C, et al. Interhemispheric resting-state functional connectivity abnormalities in type 2 diabetes patients. Ann Palliat Med. 2021;10:8123–33. 34353097 10.21037/apm-21-1655

[106] Li Y, Li M, Feng Y, Ma X, Tan X, Chen Y, et al. Aberrant brain spontaneous activity and synchronization in type 2 diabetes mellitus subjects without mild cognitive impairment. Front Neurosci. 2021;15:749730. 34975372 10.3389/fnins.2021.749730 PMC8716545

[107] Cui Y, Liang X, Gu H, Hu Y, Zhao Z, Yang X-Y, et al. Cerebral perfusion alterations in type 2 diabetes and its relation to insulin resistance and cognitive dysfunction. Brain Imaging Behav. 2017;11:1248–57. 27714551 10.1007/s11682-016-9583-9 PMC5653700

[108] Dai W, Duan W, Alfaro FJ, Gavrieli A, Kourtelidis F, Novak V. The resting perfusion pattern associates with functional decline in type 2 diabetes. Neurobiol Aging. 2017;60:192–202. 28992987 10.1016/j.neurobiolaging.2017.09.004 PMC5687828

[109] Ferreira FS, Pereira JMS, Reis A, Sanches M, Duarte JV, Gomes L, et al. Early visual cortical structural changes in diabetic patients without diabetic retinopathy. Graefes Arch Clin Exp Ophthalmol. 2017;255:2113–8. 28779362 10.1007/s00417-017-3752-4

[110] Gao Y, Sui C, Chen B, Xin H, Che Y, Zhang X, et al. Voxel-based morphometry reveals the correlation between gray matter volume and serum P-tau-181 in type 2 diabetes mellitus patients with different HbA1c levels. Front Neurosci. 2023;17:1202374. 37255749 10.3389/fnins.2023.1202374 PMC10225590

[111] Hajek T, Calkin C, Blagdon R, Slaney C, Uher R, Alda M. Insulin resistance, diabetes mellitus, and brain structure in bipolar disorders. Neuropsychopharmacology. 2014;39:2910–8. 25074491 10.1038/npp.2014.148 PMC4200504

[112] Zhang D, Lei Y, Gao J, Qi F, Yan X, Ai K, et al. Right frontoinsular cortex: a potential imaging biomarker to evaluate T2DM-induced cognitive impairment. Front Aging Neurosci. 2021;13:674288. 34122050 10.3389/fnagi.2021.674288 PMC8193040

[113] Zhang J, Liu Y, Guo X, Guo J, Du Z, He M, et al. Causal structural covariance network suggesting structural alterations progression in type 2 diabetes patients. Front Hum Neurosci. 2022;16:936943. 35911591 10.3389/fnhum.2022.936943 PMC9336220

[114] Oh DJ, Jung J-J, Shin SA, Kim H, Park S, Sohn BK, et al. Brain structural alterations, diabetes biomarkers, and cognitive performance in older adults with dysglycemia. Front Neurol. 2021;12:766216. 34777234 10.3389/fneur.2021.766216 PMC8581483

[115] Redel JM, DiFrancesco M, Vannest J, Altaye M, Beebe D, Khoury J, et al. Brain gray matter volume differences in obese youth with type 2 diabetes: a pilot study. J Pediatr Endocrinol Metab. 2018;31:261–8. 29373319 10.1515/jpem-2017-0349

[116] Moran C, Phan TG, Chen J, Blizzard L, Beare R, Venn A, et al. Brain atrophy in type 2 diabetes: regional distribution and influence on cognition. Diabetes Care. 2013;36:4036–42. 23939539 10.2337/dc13-0143 PMC3836136

[117] García-Casares N, Berthier ML, Jorge RE, Gonzalez-Alegre P, Gutiérrez Cardo A, Rioja Villodres J, et al. Structural and functional brain changes in middle-aged type 2 diabetic patients: a cross-sectional study. J Alzheimers Dis. 2014;40:375–86. 24448784 10.3233/JAD-131736

[118] Liu D, Duan S, Zhang J, Zhang Y, Wei P, Wang J. Subbands analysis of amplitude of low-frequency fluctuations in type 2 diabetes mellitus patients: a resting-state functional MRI study. Chin J Radiol. 2015;49:801–6.

[119] Zhang Q, Ding H. Meta-analysis of resting-state fMRI in cervical spondylosis patients using AES-SDM. Front Neurol. 2024;15:1439939. 39381074 10.3389/fneur.2024.1439939 PMC11460301

[120] Macpherson H, Formica M, Harris E, Daly RM. Brain functional alterations in type 2 diabetes - a systematic review of fMRI studies. Front Neuroendocrinol. 2017;47:34–46. 28687473 10.1016/j.yfrne.2017.07.001

[121] Peng J, Qu H, Peng J, Luo T-Y, Lv F-J, Chen L, et al. Abnormal spontaneous brain activity in type 2 diabetes with and without microangiopathy revealed by regional homogeneity. Eur J Radiol. 2016;85:607–15. 26860674 10.1016/j.ejrad.2015.12.024

[122] Henderson D, Bichoutar I, Moxham B, Faidherbe V, Plaisant O, Guédon A. Descriptive and functional anatomy of the Heschl Gyrus: historical review, manual labelling and current perspectives. Surg Radiol Anat. 2023;45:337–50. 36859607 10.1007/s00276-023-03114-x

[123] Li Y-L, Wu J-J, Li W-K, Gao X, Wei D, Xue X, et al. Effects of individual metabolic brain network changes co-affected by T2DM and aging on the probabilities of T2DM: protective and risk factors. Cereb Cortex. 2024;34:bhad439. 37991271 10.1093/cercor/bhad439

[124] Hullett PW, Hamilton LS, Mesgarani N, Schreiner CE, Chang EF. Human superior temporal gyrus organization of spectrotemporal modulation tuning derived from speech stimuli. J Neurosci. 2016;36:2014–26. 26865624 10.1523/JNEUROSCI.1779-15.2016 PMC4748082

[125] Allen KV, Pickering MJ, Zammitt NN, Hartsuiker RJ, Traxler MJ, Frier BM, et al. Effects of acute hypoglycemia on working memory and language processing in adults with and without type 1 diabetes. Diabetes Care. 2015;38:1108–15. 25758768 10.2337/dc14-1657 PMC4876671

[126] Sutoko S, Atsumori H, Obata A, Funane T, Kandori A, Shimonaga K, et al. Lesions in the right Rolandic operculum are associated with self-rating affective and apathetic depressive symptoms for post-stroke patients. Sci Rep. 2020;10:20264. 33219292 10.1038/s41598-020-77136-5 PMC7679372

[127] Wei Q, Lin S-M, Xu S-H, Zou J, Chen J, Kang M, et al. Graph theoretical analysis and independent component analysis of diabetic optic neuropathy: a resting-state functional magnetic resonance imaging study. CNS Neurosci Ther. 2024;30:e14579. 38497532 10.1111/cns.14579 PMC10945884

[128] Carlén M. What constitutes the prefrontal cortex? Science. 2017;358:478–82. 29074767 10.1126/science.aan8868

[129] Chen Y, Liu Z, Zhang J, Xu K, Zhang S, Wei D, et al. Altered brain activation patterns under different working memory loads in patients with type 2 diabetes. Diabetes Care. 2014;37:3157–63. 25404661 10.2337/dc14-1683

[130] Fang F, Lai M-Y, Huang J-J, Kang M, Ma M-M, Li K-A, et al. Compensatory hippocampal connectivity in young adults with early-stage type 2 diabetes. J Clin Endocrinol Metab. 2019;104:3025–38. 30817818 10.1210/jc.2018-02319

[131] Huang Y, Zhang X, Cheng M, Yang Z, Liu W, Ai K, et al. Altered cortical thickness-based structural covariance networks in type 2 diabetes mellitus. Front Neurosci. 2024;18:1327061. 38332862 10.3389/fnins.2024.1327061 PMC10851426

[132] Morgane PJ, Galler JR, Mokler DJ. A review of systems and networks of the limbic forebrain/limbic midbrain. Prog Neurobiol. 2005;75:143–60. 15784304 10.1016/j.pneurobio.2005.01.001

[133] García-Casares N, Jorge RE, García-Arnés JA, Acion L, Berthier ML, Gonzalez-Alegre P, et al. Cognitive dysfunctions in middle-aged type 2 diabetic patients and neuroimaging correlations: a cross-sectional study. J Alzheimers Dis. 2014;42:1337–46. 25024335 10.3233/JAD-140702

[134] Van Overwalle F, Baetens K, Mariën P, Vandekerckhove M. Social cognition and the cerebellum: a meta-analysis of over 350 fMRI studies. Neuroimage. 2014;86:554–72. 24076206 10.1016/j.neuroimage.2013.09.033

[135] Huang X, Xie B-J, Qi C-X, Tong Y, Shen Y. Abnormal intrinsic functional network hubs in diabetic retinopathy patients. Neuroreport. 2021;32:498–506. 33657077 10.1097/WNR.0000000000001620

[136] Zhang D, Gao J, Yan X, Tang M, Zhe X, Cheng M, et al. Altered functional connectivity of brain regions based on a meta-analysis in patients with T2DM: a resting-state fMRI study. Brain Behav. 2020;10:e01725. 32558376 10.1002/brb3.1725 PMC7428490

[137] Liu J, Yang X, Li Y, Xu H, Ren J, Zhou P. Cerebral blood flow alterations in type 2 diabetes mellitus: a systematic review and meta-analysis of arterial spin labeling studies. Front Aging Neurosci. 2022;14:847218. 35250549 10.3389/fnagi.2022.847218 PMC8888831

[138] Dupont P, Orban GA, De Bruyn B, Verbruggen A, Mortelmans L. Many areas in the human brain respond to visual motion. J Neurophysiol. 1994;72:1420–4. 7807222 10.1152/jn.1994.72.3.1420

[139] Barbier EL, Marrett S, Danek A, Vortmeyer A, van Gelderen P, Duyn J, et al. Imaging cortical anatomy by high-resolution MR at 3.0T: detection of the stripe of Gennari in visual area 17. Magn Reson Med. 2002;48:735–8. 12353293 10.1002/mrm.10255

[140] Tan X, Liang Y, Zeng H, Qin C, Li Y, Yang J, et al. Altered functional connectivity of the posterior cingulate cortex in type 2 diabetes with cognitive impairment. Brain Imaging Behav. 2019;13:1699–707. 30612339 10.1007/s11682-018-0017-8

[141] Liu L, Li W, Zhang Y, Qin W, Lu S, Zhang Q. Weaker functional connectivity strength in patients with type 2 diabetes mellitus. Front Neurosci. 2017;11:390. 28736516 10.3389/fnins.2017.00390 PMC5500656

[142] Petersen SE, Posner MI. The attention system of the human brain: 20 years after. Annu Rev Neurosci. 2012;35:73–89. 22524787 10.1146/annurev-neuro-062111-150525 PMC3413263

[143] Liu Y, Bengson J, Huang H, Mangun GR, Ding M. Top-down modulation of neural activity in anticipatory visual attention: control mechanisms revealed by simultaneous EEG-fMRI. Cereb Cortex. 2016;26:517–29. 25205663 10.1093/cercor/bhu204 PMC4712792

[144] He X-S, Wang Z-X, Zhu Y-Z, Wang N, Hu X, Zhang D-R, et al. Hyperactivation of working memory-related brain circuits in newly diagnosed middle-aged type 2 diabetics. Acta Diabetol. 2015;52:133–42. 24993663 10.1007/s00592-014-0618-7 PMC4416650

[145] Procyk E, Fontanier V, Sarazin M, Delord B, Goussi C, Wilson CRE. The midcingulate cortex and temporal integration. Int Rev Neurobiol. 2021;158:395–419. 33785153 10.1016/bs.irn.2020.12.004

[146] Chen Y-C, Xia W, Qian C, Ding J, Ju S, Teng G-J. Thalamic resting-state functional connectivity: disruption in patients with type 2 diabetes. Metab Brain Dis. 2015;30:1227–36. 26116166 10.1007/s11011-015-9700-2

[147] Chen Z, Zang X, Liu M, Liu M, Li J, Gu Z, et al. Abnormal alterations of cortical thickness in 16 patients with type 2 diabetes mellitus: a pilot MRI study. Chin Med Sci J. 2017;32:75–72. 28693687 10.24920/J1001-9294.2017.010

[148] Menon V. 20 years of the default mode network: a review and synthesis. Neuron. 2023;111:2469–87. 37167968 10.1016/j.neuron.2023.04.023 PMC10524518

[149] Chen Y, Liu Z, Wang A, Zhang J, Zhang S, Qi D, et al. Dysfunctional organization of default mode network before memory impairments in type 2 diabetes. Psychoneuroendocrinology. 2016;74:141–8. 27611859 10.1016/j.psyneuen.2016.08.012

[150] Zhang Y, Du X, Qin W, Fu Y, Wang Z, Zhang Q. Association between gene expression and altered resting-state functional networks in type 2 diabetes. Front Aging Neurosci. 2023;15:1290231. 38094506 10.3389/fnagi.2023.1290231 PMC10716229

[151] Liu D, Chen L, Duan S, Yin X, Yang W, Shi Y, et al. Disrupted balance of long- and short-range functional connectivity density in type 2 diabetes mellitus: a resting-state fMRI study. Front Neurosci. 2018;12:875. 30538618 10.3389/fnins.2018.00875 PMC6277540

[152] Jiang W, Lei Y, Wei J, Yang L, Wei S, Yin Q, et al. Alterations of interhemispheric functional connectivity and degree centrality in cervical dystonia: a resting-state fMRI study. Neural Plast. 2019;2019:7349894. 31178903 10.1155/2019/7349894 PMC6507243

[153] Zhang D, Wang M, Gao J, Huang Y, Qi F, Lei Y, et al. Altered functional connectivity of insular subregions in type 2 diabetes mellitus. Front Neurosci. 2021;15:676624. 34220433 10.3389/fnins.2021.676624 PMC8242202

[154] Liu T, Bai Y, Ma L, Ma X, Wei W, Zhang J, et al. Altered effective connectivity of bilateral hippocampus in type 2 diabetes mellitus. Front Neurosci. 2020;14:657. 32655364 10.3389/fnins.2020.00657 PMC7325692

[155] Zhang Y, Cao Y, Xie Y, Liu L, Qin W, Lu S, et al. Altered brain structural topological properties in type 2 diabetes mellitus patients without complications. J Diabetes. 2019;11:129–38. 30039563 10.1111/1753-0407.12826

[156] Lu W, Shen D, Qiu S. Association between visit-to-visit glucose variability and brain morphology and cognitive function in type 2 diabetes. J Clin Endocrinol Metab. 2025;dgaf228. 10.1210/clinem/dgaf228. 10.1210/clinem/dgaf228 PMC12623016 40198816

[157] Zhang Y, Zhang X, Ma G, Qin W, Yang J, Lin J, et al. Neurovascular coupling alterations in type 2 diabetes: a 5-year longitudinal MRI study. BMJ Open Diabetes Res Care. 2021;9:e001433. 33462074 10.1136/bmjdrc-2020-001433 PMC7816934

[158] Liang Y, Zhang H, Tan X, Liu J, Qin C, Zeng H, et al. Local diffusion homogeneity provides supplementary information in T2DM-related WM microstructural abnormality detection. Front Neurosci. 2019;13:63. 30792623 10.3389/fnins.2019.00063 PMC6374310

[159] Abbott CA, Malik RA, van Ross ERE, Kulkarni J, Boulton AJM. Prevalence and characteristics of painful diabetic neuropathy in a large community-based diabetic population in the U.K. Diabetes Care. 2011;34:2220–4. 21852677 10.2337/dc11-1108 PMC3177727

[160] Lin Y, Zhou J, Sha L, Li Y, Qu X, Liu L, et al. Metabolite differences in the lenticular nucleus in type 2 diabetes mellitus shown by proton MR spectroscopy. AJNR Am J Neuroradiol. 2013;34:1692–6. 23598834 10.3174/ajnr.A3492 PMC7965635

[161] Jiang W-H, Liu J, Zhou J, Wu Q, Pu X-Y, Chen H-H, et al. Altered dynamic brain activity and functional connectivity in thyroid-associated ophthalmopathy. Hum Brain Mapp. 2023;44:5346–56. 37515416 10.1002/hbm.26437 PMC10543102

[162] Tahamata VM, Fan Y-T, Wei L, Lin Y-N, Martinez RM, Goh KK, et al. Domain-specific and domain-general functional brain alterations in type 2 diabetes mellitus: a task-based and functional connectivity meta-analysis. Brain Behav. 2025;15:e70438. 40165500 10.1002/brb3.70438 PMC11959097

[163] Faber Benítez P, Verdejo J, León P, Reimerink A, Guzmán G. Neural substrates of specialized knowledge representation: an fMRI study. Rev Franç Linguist Appl. 2014;XIX:15–32.

[164] Gallagher HL, Frith CD. Functional imaging of ‘theory of mind’. Trends Cogn Sci. 2003;7:77–83. 12584026 10.1016/s1364-6613(02)00025-6

[165] Herold D, Spengler S, Sajonz B, Usnich T, Bermpohl F. Common and distinct networks for self-referential and social stimulus processing in the human brain. Brain Struct Funct. 2016;221:3475–85. 26365506 10.1007/s00429-015-1113-9

[166] Hu B, Rao J, Li X, Cao T, Li J, Majoe D, et al. Emotion regulating attentional control abnormalities in major depressive disorder: an event-related potential study. Sci Rep. 2017;7:13530. 29051523 10.1038/s41598-017-13626-3 PMC5648876

[167] Boller B, Mellah S, Ducharme-Laliberté G, Belleville S. Relationships between years of education, regional grey matter volumes, and working memory-related brain activity in healthy older adults. Brain Imaging Behav. 2017;11:304–17. 27734304 10.1007/s11682-016-9621-7

[168] Kocsis K, Holczer A, Kazinczi C, Boross K, Horváth R, Németh LV, et al. Voxel-based asymmetry of the regional gray matter over the inferior temporal gyrus correlates with depressive symptoms in medicated patients with major depressive disorder. Psychiatry Res Neuroimaging. 2021;317:111378. 34479177 10.1016/j.pscychresns.2021.111378

[169] Chen Y-H, Dammers J, Boers F, Leiberg S, Edgar JC, Roberts TPL, et al. The temporal dynamics of insula activity to disgust and happy facial expressions: a magnetoencephalography study. Neuroimage. 2009;47:1921–8. 19442746 10.1016/j.neuroimage.2009.04.093

[170] Choi KS, Hwang I, Moon JH, Kim M. Progressive reduction in basal ganglia explains and predicts cerebral structural alteration in type 2 diabetes. J Cereb Blood Flow Metab. 2023;43:2096–104. 37632261 10.1177/0271678X231197273 PMC10925861

[171] Hirabayashi N, Hata J, Furuta Y, Ohara T, Shibata M, Hirakawa Y, et al. Association between diabetes and gray matter atrophy patterns in a general older Japanese population: the hisayama study. Diabetes Care. 2022;45:1364–71. 35500069 10.2337/dc21-1911

[172] Vidal-Piñeiro D, Martin-Trias P, Arenaza-Urquijo EM, Sala-Llonch R, Clemente IC, Mena-Sánchez I, et al. Task-dependent activity and connectivity predict episodic memory network-based responses to brain stimulation in healthy aging. Brain Stimul. 2014;7:287–96. 24485466 10.1016/j.brs.2013.12.016 PMC4517193

[173] Turker S, Kuhnke P, Schmid FR, Cheung VKM, Weise K, Knoke M, et al. Adaptive short-term plasticity in the typical reading network. Neuroimage. 2023;281:120373. 37696425 10.1016/j.neuroimage.2023.120373 PMC10577446

[174] Lin Y, Jiang W-J, Shan P-Y, Lu M, Wang T, Li R-H, et al. The role of repetitive transcranial magnetic stimulation (rTMS) in the treatment of cognitive impairment in patients with Alzheimer’s disease: a systematic review and meta-analysis. J Neurol Sci. 2019;398:184–91. 30735817 10.1016/j.jns.2019.01.038

[175] Begemann MJ, Brand BA, Ćurčić-Blake B, Aleman A, Sommer IE. Efficacy of non-invasive brain stimulation on cognitive functioning in brain disorders: a meta-analysis. Psychol Med. 2020;50:2465–86. 33070785 10.1017/S0033291720003670 PMC7737055

[176] Tao X, Jiang M, Liu Y, Hu Q, Zhu B, Hu J, et al. Predicting three-month fasting blood glucose and glycated hemoglobin changes in patients with type 2 diabetes mellitus based on multiple machine learning algorithms. Sci Rep. 2023;13:16437. 37777593 10.1038/s41598-023-43240-5 PMC10543442

[177] Marcos-Zambrano LJ, Karaduzovic-Hadziabdic K, Loncar Turukalo T, Przymus P, Trajkovik V, Aasmets O, et al. Applications of machine learning in human microbiome studies: a review on feature selection, biomarker identification, disease prediction and treatment. Front Microbiol. 2021;12:634511. 33737920 10.3389/fmicb.2021.634511 PMC7962872

