# Supplementary material for: Structural and functional brain abnormal alteration in patients with type 2 diabetes mellitus: A coordinate-based meta-analysis
Source: Transl Psychiatry. 2025 Aug 6;15:269. doi: 10.1038/s41398-025-03488-z (PMC12328820; doi:10.1038/s41398-025-03488-z)
Supplement: Supplementary file 1 — Supplementary Materials [file 41398_2025_3488_MOESM1_ESM.docx]

**Supplementary Materials**

| **Table S1** Criteria for objective assessment of methodological quality of individual studies |
| --- |
| **Category 1: Sample characteristics (10)** |
| Patients were evaluated with specific standardized diagnostic criteria (1) |
| Important demographic data (age and gender) were reported with mean (or median) and standard deviations (or range)) (2) |
| Healthy comparison subjects were evaluated to exclude psychiatric and medical illnesses and demographic data was reported (1) |
| Important clinical variables (e.g. illness duration, medication status, HAMA scores, HAMD scores) were reported with mean (or median) and standard deviations (or range)) (4) |
| Sample size per group > 10 (2) |
| **Category 2: Methodology and reporting (10)** |
| Whole brain analysis was automated with no a-priori regional selection (3) |
| Magnet strength at least 1.5T (1) |
| At least 5 minutes of resting state acquisition (1) |
| Whole brain coverage of resting scans (1) |
| The acquisition and preprocessing techniques were clearly described so that they could be reproduced (1) |
| Coordinates reported in a standard space (1) |
| Significant results are reported after correction for multiple testing using a standard statistical procedure (FDR, FWE or permutation-based methods) (1) |
| Conclusions were consistent with the results obtained and the limitations were discussed (1) |

A maximum score of 20 for each study, allocated as per the criteria specified above. Each of the two sections, sample characteristics, methodology, and reporting, is worth 10 points, and the value following each item represents the score that the item would have received if it had met the requirements. Take the first project as an example, patients were assessed in a study using specific standardized diagnostic criteria, then the study would receive a score of 1.

**Table S2** Demographic and clinical characteristics of patients with T2DM and HCs in local metrics meta-analysis

| Study | Indices | Subjects (female) | | Mean age (SD) | | Scanner | FWHM | Software | Statistical threshold | Quality scores (out of 20)^a^ |
| --- | --- | --- | --- | --- | --- | --- | --- | --- | --- | --- |
|  |  | Patients | HC | Patients | HC |  |  |  |  |  |
| Qian et al. (2021) | ALFF | 22 (10) | 39 (16) | 59.56 (7.56) | 58.34 (6.69) | 3.0T | 4mm | DPABI | TFCE (*p*<.05) | 20 |
| Bao et al. (2022) | fALFF | 18 (2) | 18 (16) | 62.50 (3.65) | 71.33 (5.64) | 3.0T | 4mm | DPARSF, SPM | GRF (*p*<.01) | 19 |
| Wang et al. (2017) | fALFF | 17 (5) | 17 (5) | 53.4 (5.9) | 54.4 (7.9) | 3.0T | 6mm | DPARSF, SPM | Alphasim (*p*<.05) | 18 |
| Qin et al. (2021) | ReHo | 22 (10) | 31 (13) | 62 (5) | 60 (8) | 3.0T | 6mm | DPABI | TFCE (*p*<.05) | 19 |
| Shi et al. (2020) | ALFF | 31 (15) | 31 (15) | 56.03 (4.61) | 56.48 (4.29) | 3.0T | NA | DPARSF, SPM | Alphasim (*p*<.05) | 19 |
| Cui et al. (2014) | ReHo | 29 (15) | 27 (16) | 58.3 (7.3) | 57.8 (5.9) | 3.0T | NA | REST | Alphasim (*p*<.01) | 20 |
| Liu et al. (2022) | ReHo | 14 (10) | 13 (8) | 58.33 (4.63) | 56.00 (4.22) | 3.0T | 6mm | DPABI, SPM | FDR (*p*<.01) | 19 |
| Chen et al. (2022) | ReHo | 31 (NA) | 31 (NA) | 43.48 (9.7) | 41.94 (7.73) | 3.0T | 6mm | DPABI, REST | Alphasim (*p*<.05) | 19 |
| Xiong et al. (2020) | ReHo | 25 (14) | 27 (15) | 62.68 (5.65) | 59.08 (6.35) | 3.0T | NA | DPABI, SPM | Alphasim (*p*<.01) | 20 |
| Xin et al. (2023) | ALFF | 43 (13) | 32 (22) | 57.93 (7.41) | 56.94 (8.90) | 3.0T | 4mm | SPM, DPABI | GRF (*p*<.001) | 20 |
| Liu et al. (2019) | ReHo | 26 (NA) | 26 (NA) | 51.9 (10.7) | 48.2 (6.7) | 3.0T | 8mm | DPARSF, SPM | Alphasim (*p*<.01) | 20 |
| Xiang et al. (2024) | fALFF | 30 (0) | 31 (0) | 44.30 (8.02) | 41.93 (7.72) | 3.0T | 4mm | DPABI, REST | GRF (*p*<.001) | 20 |
| Xia et al. (2013) | ALFF | 28 (13) | 29 (16) | 58.7 (8.1) | 57.7 (7.2) | 3.0T | 4mm | REST, SPM | Alphasim (*p*<.05) | 20 |
| Zhou et al. (2014) | ALFF | 14 (8) | 17 (7) | 63.5 (6.91) | 63.8 (5.79) | 3.0T | 8mm | DPARSF, SPM | Monte Carlo simulations (*p*<.05) | 20 |
| Wang et al. (2017) | ALFF | 21 (11) | 16 (9) | 54.9 (9.9) | 54.8 (5.7) | 3.0T | 8mm | DPARSF, REST, SPM | Bonferroni (p<0.01) | 19 |
| Peng et al. (2016) | ReHo | 22 (12) | 28 (16) | 58.8 (7.9) | 56.2 (6.9) | 3.0T | 4mm | DPARSF, REST, SPM | Alphasim (*p*<.05) | 20 |
| Li et al. (2024) | ALFF | 40 (23) | 55 (27) | 51.50 (8.83) | 48.29 (7.30) | 3.0T | 6mm | DPABI, SPM | GRF (*p*<.001) | 20 |
| Li et al. (2021) | ALFF | 30 (NA) | 30 (NA) | 49.23 (5.52) | 45.77 (6.43) | 3.0T | 6mm | RESTplus | GRF (*p*<.01) | 19 |
| Liu et al. (2020) | ALFF | 37 (13) | 37 (20) | 57.57 (7.12) | 57.86 (5.68) | 3.0T | 4mm | DPABI | GRF (*p*<.05) | 19 |
| Liu et al. (2016) | ReHo | 25 (8) | 25 (12) | 52.24 (4.78) | 52.08 (3.46) | 3.0T | 4mm | DPARSF, SPM | Alphasim (*p*<.01) | 20 |
| Wang et al. (2014) | ALFF | 26 (NA) | 26 (NA) | NA | NA | 3.0T | 4mm | DPARSF, SPM | Monte Carlo simulations (*p*<.05) | 19 |
| Zhang et al. (2018) | fALFF | 19 (7) | 15 (7) | 53.8 (8.1) | 53.9 (5.4) | 3.0T | 3mm | DPARSF, REST | GRF (*p*<.01) | 20 |

Note: The number in brackets below the subjects means that sex is the number of females. a The quality scores of each included study were assessed by a 20-point checklist which mainly consisted of 13 items. Each study was evaluated by the items in the checklist and the study would obtain the corresponding scores in an item if it was qualified. The total score obtained from all the items represents the quality of the study.
Abbreviations: DPABI, A toolbox for data processing & processing & analysis for brain Abbreviations; DPARSF, Data processing assistant for resting-state fMRI; REST, resting-state fMRI data analysis toolkit; RESTplus, resting-state fMRI data analysis toolkit plus; SPM, statistical parametric mapping; ALFF, the amplitude of low-frequency fluctuations; fALFF, fractional amplitude of low-frequency fluctions; ReHo, regional homogeneity; FDR, false discovery rate; GRF, Gaussian random fields; TFCE, threshold free cluster enhancement; FWHM, full width at half maximum; SD, standard deviation; HC, healthy controls; NA, not applicable.

| **Table S3** Demographic and clinical characteristics of patients with T2DM and HCs in DMN-FC meta-analysis | | | | | | | | | | | |  |
| --- | --- | --- | --- | --- | --- | --- | --- | --- | --- | --- | --- | --- |
| Study | Subjects (female) | | Mean age (SD) | | Scanner | FWHM | Software | Statistical threshold | Method of analysis | Seed region | Quality scores (out of 20)^a^ |  |
|  | Patients | HC | Patients | HC |  |  |  |  |  |  |  |  |
| Zhang et al. (2021) | 69 (43) | 69 (45) | 56.94 (9.86) | 57.35 (8.35) | 3.0T | 6mm | DPARSF, REST, SPM | Monte Carlo simulations | ICA | - | 20 |  |
|  |  |  |  |  |  |  |  | (*p*<.05) |  |  |  |  |
| Xia et al. (2020) | 44 (20) | 50 (26) | 58.57 (7.44) | 56.34 (6.35) | 3.0T | 6mm | DPABI, REST, SPM | FDR (*p*<.01) | ICA | - | 19 |  |
|  |  |  |  |  |  |  |  |  |  |  |  |  |
| Li et al. (2020) | 28 (18) | 28 (15) | 54.8 (1.2) | 53.9 (1.2) | 3.0T | NA | FreeSurfer | Bonferroni (*p*<.05) | Seed-based analysis | L PCC | 18 |  |
|  |  |  |  |  |  |  |  |  |  |  |  |  |
| Lei et al. (2021) | 44 (18) | 47 (17) | 55.2 (7.26) | 54.28 (6.90) | 3.0T | 6mm | GRETNA | FDR (*p*<.001) | ICA | - | 19 |  |
|  |  |  |  |  |  |  |  |  |  |  |  |  |
| Hoogenboom et al. (2014) | 18 (7) | 19 (8) | 56.2 (6.3) | 53.1 (6.2) | 3.0T | NA | FSL | Bonferroni (*p*<.05) | Seed-based analysis | PCC | 20 |  |
|  |  |  |  |  |  |  |  |  |  |  |  |  |
| Xia et al. (2015) | 38 (21) | 40 (19) | 56.0 (6.1) | 57.1 (7.6) | 3.0T | 6mm | SPM | FWE (*p*<.01) | Seed-based analysis | PCUN | 19 |  |
|  |  |  |  |  |  |  |  |  |  |  |  |  |
| Qin et al. (2021) | 22 (10) | 31 (13) | 62 (5) | 60 (8) | 3.0T | 6mm | DPABI | TFCE (*p*<.05) | ICA | PCC | 19 |  |
| Feng et al. (2021) | 20 (6) | 20 (13) | 36.45 (3.72) | 34.05 (4.78) | 3.0T | 8mm | DARTEL, SPM | Alphasim (*p*<.05) | Seed-based analysis | STG.L, Left Heschl gyrus, and Hip.L | 18 |  |
|  |  |  |  |  |  |  |  |  |  |  |  |  |
| Wu et al. (2022) | 82 (29) | 66 (24) | 46.09 (8.17) | 46.64 (11.03) | 3.0T | 6mm | SPM | FDR (*p*<.05) | ICA | - | 19 |  |
|  |  |  |  |  |  |  |  |  |  |  |  |  |
| Huang et al. (2022) | 31 (13) | 34 (13) | 56.09 (6.11) | 53.48 (4.82) | 3.0T | 4mm | DPABI | FDR (*p*<.05) | Seed-based analysis | R PHG | 20 |  |
|  |  |  |  |  |  |  |  |  |  |  |  |  |
| Wang et al. (2022) | 44 (13) | 41 (14) | 55.18 (6.25) | 54.41 (4.96) | 3.0T | 6mm | DPABI, SPM | GRF (*p*<.05) | Seed-based analysis | R dAI | 19 |  |
|  |  |  |  |  |  |  |  |  |  |  |  |  |
| Li et al. (2024) | 40 (23) | 55 (27) | 51.50 (8.83) | 48.29 (7.30) | 3.0T | 6mm | DPABI, SPM | GRF (*p*<.001) | Seed-based analysis | PCUN.R | 20 |  |
|  |  |  |  |  |  |  |  |  |  |  |  |  |
| Cui et al. (2015) | 44 (32) | 42 (19) | 60.4 (7.0) | 58.2 (6.3) | 3.0T | 4mm | REST, SPM | Alphasim(*p*<.05) | ICA | - | 18 |  |
|  |  |  |  |  |  |  |  |  |  |  |  |  |
| Li et al. (2021) | 30 (NA) | 30 (NA) | 49.23 (5.52) | 45.77 (6.43) | 3.0T | 6mm | RESTplus | GRF (*p*<.01) | Seed-based analysis | ORBinf.R | 19 |  |
|  |  |  |  |  |  |  |  |  |  |  |  |  |
| Liu et al. (2020) | 37 (13) | 37 (20) | 57.57 (7.12) | 57.86 (5.68) | 3.0T | 4mm | DPABI | GRF (*p*<.05) | Seed-based analysis | MOG.L | 19 |  |
|  |  |  |  |  |  |  |  |  |  |  |  |  |
| Liu et al. (2016) | 25 (8) | 25 (12) | 52.24 (4.78) | 52.08 (3.46) | 3.0T | 4mm | DPARSF, SPM | Alphasim (*p*<.01) | Seed-based analysis | ACG.R, FFG.R, PreCG.R, SFG | 20 |  |
|  |  |  |  |  |  |  |  |  |  |  |  |  |
| Lips et al. (2014) | 19 (19) | 27 (27) | 51.0 (7.1) | 47.7 (6.4) | 3.0T | 6mm | FSL | Bonferroni (*p*<.05) | Seed-based analysis | PCC | 16 |  |
|  |  |  |  |  |  |  |  |  |  |  |  |  |
| Musen et al. (2012) | 10 (3) | 11 (4) | 56 (2.2) | 54 (1.8) | 3.0T | NA | FSL | Multiple (*p*<.05) | Seed-based analysis | PCC | 19 |  |
|  |  |  |  |  |  |  |  |  |  |  |  |  |
| Yu et al. (2019) | 33 (5) | 33 (11) | 53.45 (8.4) | 51.00 (5.3) | 3.0T | 8mm | DPABI | GRF (*p*<.05) | Seed-based analysis | R Precuneus,  L STG | 15 |  |
|  |  |  |  |  |  |  |  |  |  |  |  |  |
| Note: The number in brackets below the subjects means that sex is the number of females. a The quality scores of each included study were assessed by a 20-point checklist which mainly consisted of 13 items. Each study was evaluated by the items in the checklist and the study would obtain the corresponding scores in an item if it was qualified. The total score obtained from all the items represents the quality of the study. Abbreviations: DPABI, A toolbox for data processing & processing & analysis for brain Abbreviations; DPARSF, Data processing assistant for resting-state fMRI; GRETNA, A graph theoretical network analysis toolbox for imaging connections; REST, resting-state fMRI data analysis toolkit; SPM, statistical parametric mapping; ICA, Independent Component Analysis; FDR, false discovery rate; FWE, family-wise error; GRF, Gaussian random fields; L, left; R, right; dAI, dorsal anterior insula; default mode network; FWHM, full width at half maximum; ACG, Anterior cingulate and paracingulate gyri; PCC, Posterior Cingulate Cortex; PHG, Parahippocampal gyrus; PreCG, Precental gyrus; PCUN, Precuneus; FFG, Fusiform gyrus; SFG, Superior frontal gyru; HIP, Hippocampus; MOG, Middle occipital gyrus; ORBinf, Inferior frontal gyrus, orbital part;SD, standard deviation; HC, healthy controls; NA, not applicable. | | | | | | | | | | | |  |

| **Table S4** Demographic and clinical characteristics of patients with T2DM and HCs in VBM meta-analysis | | | | | | | | | |  |
| --- | --- | --- | --- | --- | --- | --- | --- | --- | --- | --- |
| Study | Subjects (female) | | Mean age (SD) | | Scanner | FWHM | Software | Statistical threshold | Quality scores |  |
|  | Patient | HC | Patient | HC |  |  |  |  | (out of 20)^a^ |  |
| Nouwen et al. (2017) | 13 (0) | 19 (14) | 16.1 (1.6) | 16.1 (1.9) | 3.0T | 6mm | SPM, DARTEL | FWE (*p*<.05) | 19 |  |
| Chen et al. (2012) | 16 (12) | 16 (12) | 61.2 (7.8) | 59.6 (6.1) | 3.0T | 8mm | DARTEL, SPM | FDR (*p*<.05) | 18 |  |
| Hajek et al. (2014） | 33 (17) | 11 (7) | 48.3 (8.7) | 43.1 (10.4) | 3.0T | NA | SPM | TFCE (*p*<.05) | 18 |  |
| Zhang et al. (2014) | 25 (17) | 28 (18) | 52.24 (9.25) | 55.48 (9.09) | 3.0T | 8mm | SPM | Alphasim (*p*<.05) | 18 |  |
| Ferreira et al. (2017) | 24 (11) | 27 (14) | 58.58 (8.63) | 59.89 (5.93) | 3.0T | 8mm | SPM | uncorr (*p*<.001) | 17 |  |
| Wang et al.(2017) | 17 (5) | 17 (5) | 53.4 (5.9) | 54.4 (7.9) | 3.0T | 8mm | DPARSF, SPM | Alphasim (*p*<.05) | 18 |  |
| Cui et al. (2017) | 40 (19) | 41 (28) | 60.5 (6.9) | 57.9 (6.5) | 3.0T | 8mm | SPM | FWE (*p*<.05) | 18 |  |
| Zhang et al. (2022) | 81 (30) | 48 (26) | 54.15 (9.26) | 54.13 (7.50) | 3.0T | 6mm | DPARSF, SPM | GRF (*p*<.001) | 19 |  |
| Oh et al. (2021) | 23 (9) | 14 (11) | 71.3 (6.3) | 72.6 (4.2) | 3.0T | 8mm | SPM, CAT | Bonferroni (*p*<.05) | 19 |  |
| Redel et al. (2018) | 20 (3) | 20 (3) | 16.7 (2.6) | 16.7 (2.0) | 3.0T | 8mm | SPM | FWE (*p*<.05) | 19 |  |
| Chris Moran et al. (2013) | 350 (140) | 363 (168) | 67.8 (6.9) | 72.1 (7.2) | 1.5T | 8mm | NA | FDR (*p*<.001) | 19 |  |
| Feng et al. (2021) | 20 (6) | 20 (13) | 36.45 (3.72) | 34.05 (4.78) | 3.0T | 8mm | DARTEL, SPM | Alphasim (*p*<.05) | 18 |  |
| Chen et al. (2016) | 23 (11) | 24 (12) | 60.78 (8.31) | 57.00 (7.48) | 3.0T | 8mm | FSL, SPM | TFCE (*p*<.05) | 20 |  |
| [García-Casares et al. (2014)](https://pubmed.ncbi.nlm.nih.gov/?term=Garc%C3%ADa-Casares+N&cauthor_id=24448784) | | 25 (8) | 25 (11) | 60.0 (4.6) | 57.8 (5.4) | 3.0T | 6mm | SPM | Alphasim (*p*<.05) | 20 |
| Zhang et al. (2021) | 34 (14) | 31 (13) | 53.97 (7.60) | 53.42 (4.97) | 3.0T | 6mm | SPM | GRF (*p*<.001) | 19 |  |
| Dai et al. (2017) | 41 (22) | 32 (16) | 65.51 (8.30) | 67.28 (10.08) | 3.0T | 8mm | SPM | FWE (*p*<.05) | 18 |  |
| Gao et al. (2023) | 41 (20) | 42 (23) | 57.44 (9.4) | 54.19 (8.19) | 3.0T | 8mm | DPARSF, SPM | FDR (*p*<.001) | 16 |  |
| [Crisóstomo et al. (2021)](https://pubmed.ncbi.nlm.nih.gov/?term=%22Cris%C3%B3stomo%20J%22%5bAuthor%5d) | 86 (35) | 40 (20) | 60.67 (7.89) | 57.73 (8.06) | 3.0T | 12mm | SPM, CAT | FWE (*p*<.05) | 20 |  |

Note: The number in brackets below the subjects means that sex is the number of females. a The quality scores of each included study were assessed by a 20-point checklist which mainly consisted of 13 items. Each study was evaluated by the items in the checklist and the study would obtain the corresponding scores in an item if it was qualified. The total score obtained from all the items represents the quality of the study.
Abbreviations: DARTEL, Diffeomorphic anatomical registration through exponential lie algebra; DPARSF, Data processing assistant for resting-state fMRI; SPM, statistical parametric mapping; VBM, voxel-based morphometry; FDR, false discovery rate; FWE, family-wise error; FWHM, full width at half maximum; GRF, Gaussian random fields; TFCE, threshold free cluster enhancement; SD, standard deviation; HC, healthy controls; uncorr, uncorrected; NA, not applicable.

**Table S5** The results of Jackknife sensitivity analysis, heterogeneity, and publication bias by AES-SDM analysis for local metrics

| Brain regions | BA | MNI coordinates | | | Jackknife sensitivity analysis | Heterogeneity | Egger’ test *p* value |
| --- | --- | --- | --- | --- | --- | --- | --- |
|  |  | x | y | z |  |  |  |
| Right inferior cerebellum | NA | 20 | -68 | -50 | 22/22 | No | 0.860 |
| Left inferior cerebellum | NA | -42 | -50 | -52 | 20/22 | No | 0.796 |
| Left superior cerebellum | 20 | -30 | -30 | -30 | 19/22 | No | 0.045 |
| Left parahippocampal gyrus | 35 | -18 | -18 | -18 | 18/22 | No | 0.110 |
| Left precentral gyrus | 6 | -30 | -6 | 56 | 22/22 | No | 0.001 |
| Left lingual gyrus | NA | 0 | -76 | 4 | 22/22 | No | 0.412 |
| Left inferior frontal gyrus | NA | -52 | 12 | 28 | 20/22 | Yes | 0.897 |
| Left superior temporal gyrus | 42 | -52 | -34 | 18 | 19/22 | No | 0.120 |
| Right Heschl's gyrus | 48 | 54 | -8 | 6 | 17/22 | No | 0.293 |
| Left superior frontal gyrus | 9 | -20 | 30 | 48 | 19/22 | Yes | 0.792 |

Note: This AES-SDM analysis was thresholded with Voxel threshold *p*-value < .005.
Abbreviations: BA, Brodmann; L, left; R right; MNI, Montreal Neurological institute.

**Table S6** The results of Jackknife sensitivity analysis, heterogeneity and publication bias by AES-SDM analysis for DMN-FC

| Brain regions | BA | MNI coordinates | | | Jackknife sensitivity analysis | Heterogeneity | Egger’s test *p* value |
| --- | --- | --- | --- | --- | --- | --- | --- |
|  |  | x | y | z |  |  |  |
| Right superior frontal gyrus | 9 | 20 | 36 | 46 | 19/19 | Yes | 0.162 |
| Left middle occipital gyrus | 19 | -34 | -78 | 20 | 18/19 | Yes | 0.102 |
| Right superior frontal gyrus | 11 | 22 | 56 | 2 | 17/19 | Yes | 0.060 |
| Left superior parietal gyrus | 7 | -24 | -72 | 54 | 18/19 | Yes | 0.087 |
| Left precuneus | 5 | -6 | -44 | 74 | 18/19 | Yes | 0.705 |
| Left superior frontal gyrus | 10 | -14 | 58 | 14 | 17/19 | No | 0.220 |
| Left middle cingulate cortex | NA | -16 | -54 | 40 | 16/19 | No | 0.079 |
| Right precuneus | 23 | 8 | -54 | 22 | 19/19 | Yes | 0.797 |
| Left superior temporal gyrus | 48 | -54 | -20 | 14 | 18/19 | No | 0.642 |
| Left angular gyrus | 39 | -42 | -60 | 44 | 18/19 | Yes | 0.519 |
| Left anterior cingulate cortex | NA | 0 | 40 | 24 | 17/19 | Yes | 0.630 |
| Right angular gyrus | 40 | 36 | -54 | 50 | 16/19 | No | 0.570 |
| Right superior temporal gyrus | 22 | 62 | -14 | 12 | 15/19 | Yes | 0.438 |

Note: This AES-SDM analysis was thresholded with Voxel threshold *p*-value < .005.
Abbreviations: BA, Brodmann; L, left; R right; MNI, Montreal Neurological institute.

**Table S7** The results of Jackknife sensitivity analysis, heterogeneity and publication bias by AES-SDM analysis for VBM

| Brain regions | BA | MNI coordinates | | | Jackknife sensitivity analysis | Heterogeneity | Egger’s test *p* value |
| --- | --- | --- | --- | --- | --- | --- | --- |
|  |  | x | y | z |  |  |  |
| Right insula | 45 | 42 | 20 | 4 | 18/18 | Yes | 0.507 |
| Left superior cerebelum | 19 | -34 | -64 | -18 | 16/18 | No | 0.590 |
| Right superior occipital gyrus | 18 | 22 | -94 | 24 | 17/18 | Yes | 0.889 |
| Right postcentral gyrus | 3 | 58 | -8 | 34 | 15/18 | Yes | 0.557 |
| Right inferior temporal gyrus | 20 | 48 | -10 | -24 | 16/18 | No | 0.152 |
| Left precuneus | 7 | -2 | -70 | 46 | 18/18 | Yes | < 0.001 |
| Left inferior temporal gyrus | 20 | -56 | -26 | -24 | 17/18 | No | < 0.001 |
| Left putamen | 11 | -16 | 16 | -2 | 14/18 | No | 0.001 |
| Right inferior frontal gyrus | 47 | 44 | 48 | -10 | 16/18 | Yes | < 0.001 |
| Left precuneus | NA | -12 | -58 | 50 | 18/18 | Yes | < 0.001 |
| Left middle temporal gyrus | 21 | -60 | -10 | -16 | 15/18 | Yes | < 0.001 |
| Left middle temporal gyrus | 22 | -58 | -42 | 4 | 16/18 | Yes | < 0.001 |

Note: This AES-SDM analysis was thresholded with Voxel threshold *p*-value < .005.
Abbreviations: BA, Brodmann; L, left; R right; VBM, voxel-based morphometry; MNI, Montreal Neurological institute.


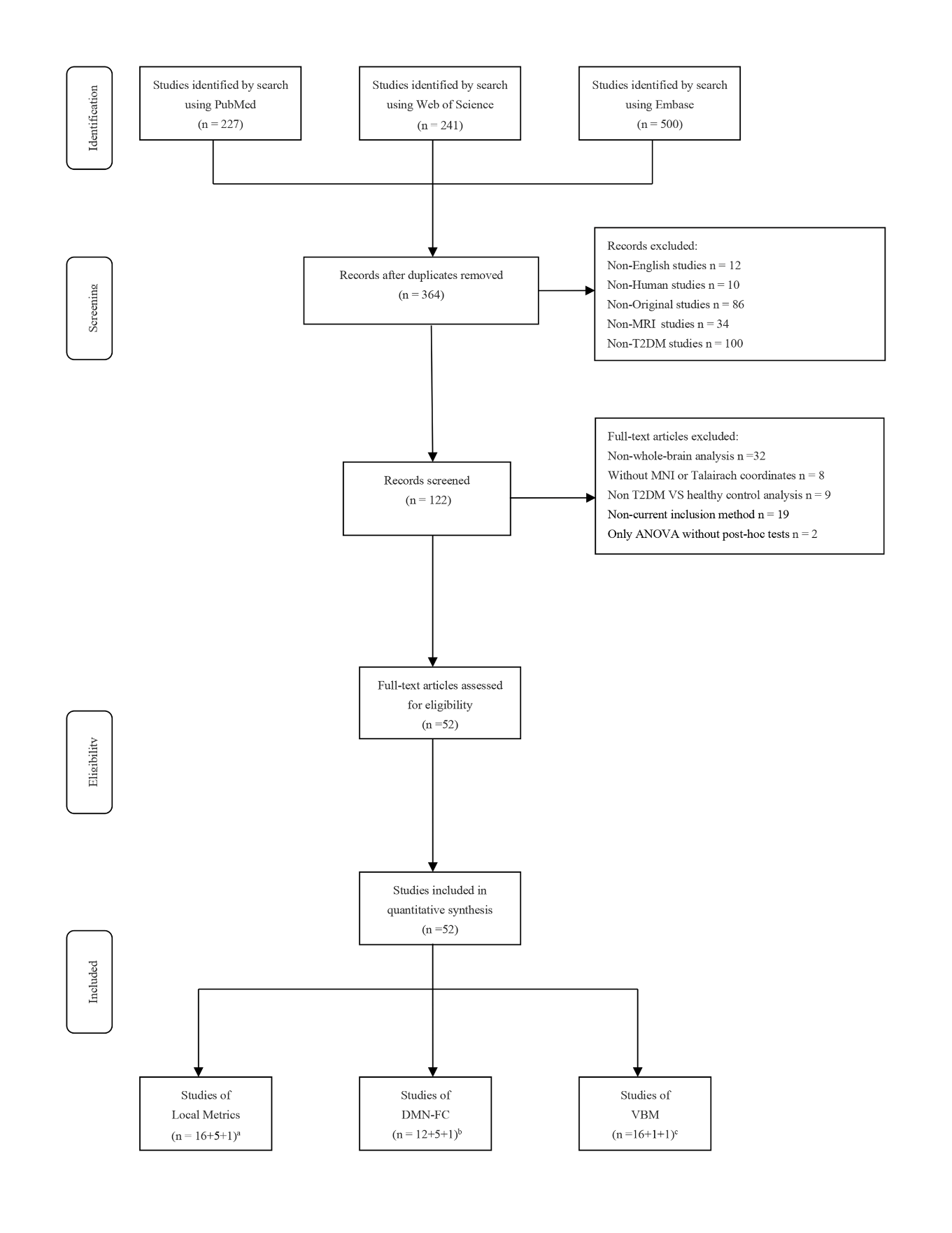


**Figure S1** Flow diagram of literature search, study inclusion and exclusion.

^a^ represents there were 16 studies of local metrics, 5 studies both including local metrics and DMN-FC analysis, 1 study both including local metrics and VBM analysis.

^b^ represents there were 12 studies of DMN-FC, 5 studies both including local metrics and DMN-FC analysis, 1 study both including DMN-FC and VBM analysis.

^c^ represents there were 16 studies of VBM, 1 study both including local metrics and VBM analysis, 1 study both including DMN-FC and VBM analysis.
